# A Guide for *Ex Vivo* Handling and Storage of Stool Samples Intended for Fecal Microbiota Transplantation

**DOI:** 10.1038/s41598-019-45173-4

**Published:** 2019-06-20

**Authors:** Sebastian D. Burz, Anne-Laure Abraham, Fernanda Fonseca, Olivier David, Audrey Chapron, Fabienne Béguet-Crespel, Stéphanie Cénard, Karine Le Roux, Orlane Patrascu, Florence Levenez, Carole Schwintner, Hervé M. Blottière, Christel Béra-Maillet, Patricia Lepage, Joël Doré, Catherine Juste

**Affiliations:** 1 Micalis Institute, INRA, AgroParisTech, Université Paris-Saclay, 78350 Jouy-en-Josas, France; 2 MaIAGE, INRA, Université Paris-Saclay, 78350 Jouy-en-Josas, France; 3 GMPA, INRA, AgroParisTech, Université Paris-Saclay, 78850 Thiverval-Grignon, France; 4 Université Paris-Saclay, MetaGenoPolis, INRA, 78350 Jouy-en-Josas, France; 5 MaaT Pharma, Pharmaceutical Development, 69007 Lyon, France

**Keywords:** Clinical microbiology, Biologics

## Abstract

Owing to the growing recognition of the gut microbiota as a main partner of human health, we are expecting that the number of indications for fecal microbiota transplantation (FMT) will increase. Thus, there is an urgent need for standardization of the entire process of fecal transplant production. This study provides a complete standardized procedure to prepare and store live and ready-to-use transplants that meet the standard requirements of good practices to applied use in pharmaceutical industry. We show that, if time before transformation to transplants would exceed 24 hours, fresh samples should not be exposed to temperatures above 20 °C, and refrigeration at 4 °C can be a safe solution. Oxygen-free atmosphere was not necessary and simply removing air above collected samples was sufficient to preserve viability. Transplants prepared in maltodextrin-trehalose solutions, stored in a -80 °C standard freezer and then rapidly thawed at 37 °C, retained the best revivification potential as  proven by 16S rRNA profiles, metabolomic fingerprints, and flow cytometry assays over a 3-month observation period. Maltodextrin-trehalose containing cryoprotectants were also efficient in preserving viability of lyophilized transplants, either in their crude or purified form, an option that can be attractive for fecal transplant biobanking and oral formulation.

## Introduction

The human intestinal microbiota ensures several essential functions that support maintenance of health and well-being. One of them is the prevention of colonization with allochtonous microbes including pathogens, also known as barrier effect or competitive exclusion. This is dependent on the maintenance of ecological homeostasis of the gut ecosystem^1^. Another well-established function of the intestinal microbiota is its contribution to immune maturation and maintenance of a balanced immune tolerance and reactivity. It is mediated by a constant and intimate crosstalk between intestinal microbiota and the gut associated lymphoid tissues^2^. Here again, a balanced microbiota-host crosstalk is dependent on the maintenance of ecological homeostasis of the gut ecosystem.

In healthy adults, microbiota composition is individual specific and for a given person, it remains fairly stable over time^3^. Upon mild stress such as exposure to specific nutrients or diverse conditions of modern daily life or clinical practices, including antibiotics, the intestinal microbiota can be temporarily altered but will eventually return to its original composition; it is resilient^4^. Nonetheless, marked alterations of dietary habits, environmental exposures, drug therapy or surgical interventions will induce alterations of the intestinal microbiota that may exceed the resilience capacity^5,6^ and lead to onset of dysbiosis, i.e. distorted intestinal microbiota composition. Dysbiosis is now acknowledged as a common feature of numerous non-infectious diseases of modern societies, which have had a constant increase in incidence since the middle of the past century. Obesity is one of these, now recognized by WHO as a world epidemic^7^. Others include inflammatory bowel diseases^8^, cardio-metabolic diseases^9^, allergies^10^ and cancers^11^.

At the same time, molecular tools for the assessment of the intestinal microbiota have made tremendous progress such that predictive/prognostic signatures may be identified in many contexts^12–15^. In turn, for most of these diseases, scientific observations highlight the existence of dysbiosis, calling for modulations of the microbiota as curative or preventive measures^16^.

As microbiota can be modulated by nutrition, drugs or FMT, beneficial functions dependent on intestinal ecological homeostasis can most likely be promoted or restored^17^. The ability of FMT to cure patients from recurrent *Clostridioides* (formely *Clostridium*) *difficile* infection (rCDI) has been recently revisited so it is becoming the recommended practice in this particular clinical condition^18–20^. Overall, FMT has gained visibility and renewed interest in the clinical domain^21^ over the past decade and it may be relevant for disease-associated microbiota alterations, and clinical practice- or daily-life-induced alterations.

However, in this domain, current practice is still essentially empirical, benefiting from half a century of compassionate practice and disparate trials, but unfortunately without a sound knowledge of key determinants of success, nor ecological impact in the long run. In this context, only minimum guidelines exist for the preparation and conservation of fecal transplants, and they are ‘based on what has been described, but never rigorously tested’^20^.

One exception to this statement is a bench to bedside study published two years ago^22^ that tested a broad range of cryoconservation agents and holding conditions for encapsulated freeze-dried fecal transplants, whose engraftment was tested in both a mouse model of CDI and human rCDI patients. The selected product was a freeze-dried fecal suspension in 5% trehalose that retained 56% intact cells, a percentage indistinguishable from fresh material.

In the present paper, we focused on the different environmental factors that may have an impact on the microbial content and viability of stool samples before and after their transformation to transplants. Time, temperature and atmosphere conditions, as well as new freeze-dryable solutions are addressed for collecting, conditioning, storing and reconditioning intestinal samples intended for FMT. Microbial taxonomic (16S rRNA) and metabolomic fingerprints are used for *ex vivo* in-depth examination of a limited number of ready-to-use transplants. The ability of the microbial communities to survive to different handling and storage conditions is addressed by their growing capacity when placed in a dedicated culture broth. We also propose an easy and convenient live/dead test by flow cytometry for routine analysis of bacterial viability in fecal transplants at different steps of the process.

## Results

### Impact of short-term storage conditions on raw samples

**Figure 1:**
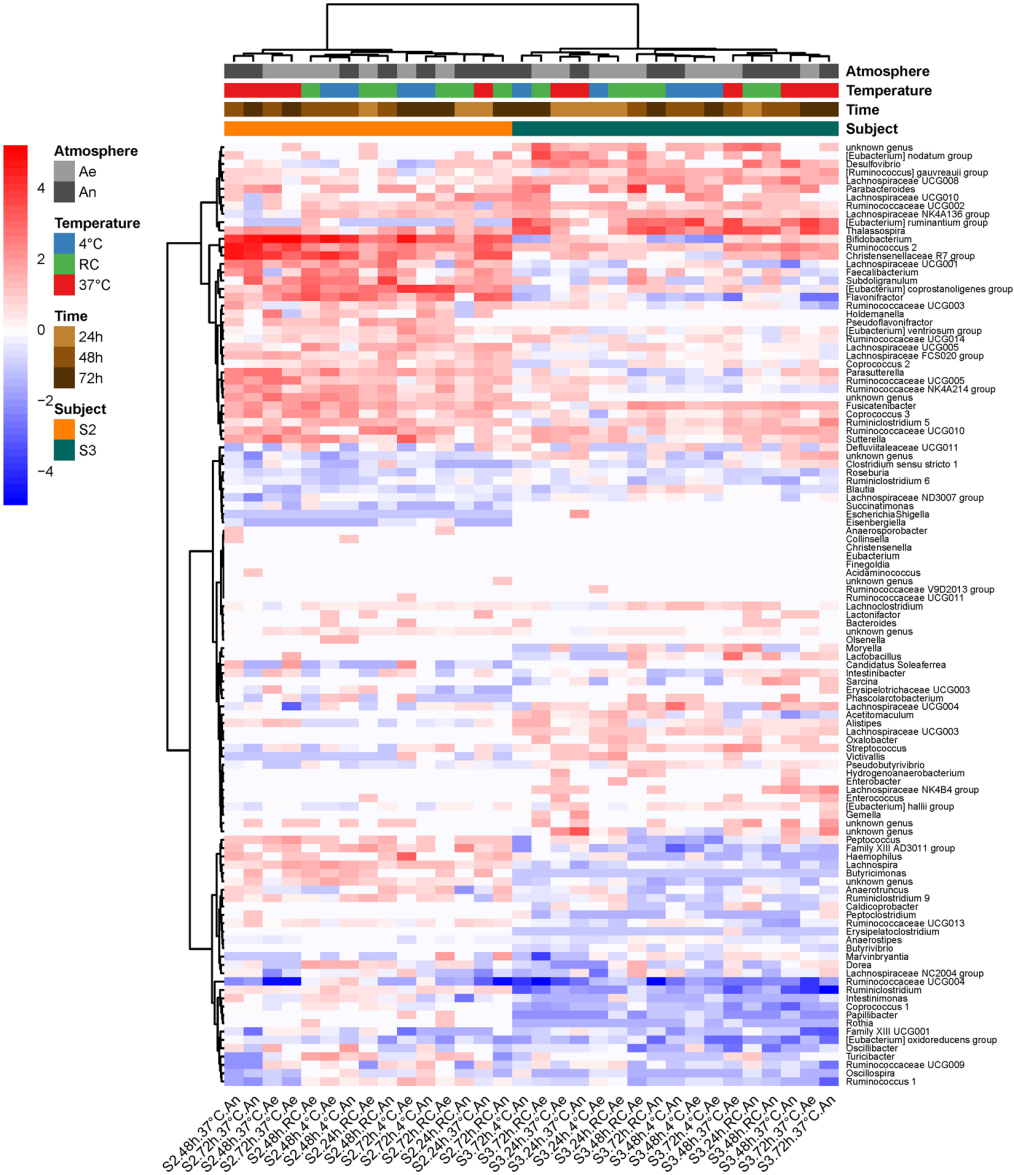
Heatmap of log2(condition/baseline) for genus abundance in raw fecal samples from two individuals (S2 and S3). Conditions are combinations of temperature (37 °C, RC for room temperature, and 4 °C), atmosphere (An and Ae for anaerobiosis and aerobiosis, respectively), and time after stool collection (24, 48 and 72 hours). Baseline is genus abundance within two hours of stool collection.

We examined the microbial taxonomic (16S rRNA) and metabolomic fingerprints of two individual stool donations (S2, S3, Supplementary Fig. S1), which were left 24, 48 or 72 hours at three temperatures (2–6 °C, 20 ± 2 °C or 37 °C) under aerobiosis or anaerobiosis. We computed Bray-Curtis dissimilarities from baseline (freshly collected samples) in order to identify relative abundance changes in Operational Taxonomic Units (OTUs) composition. A two-way ANOVA showed no main effect of either independent variable (time, donor, temperature or atmosphere), but a significant two-way interaction temperature:donor on the one hand, and atmosphere:donor on the other (Table 1), indicating that the effect of temperature and atmosphere differed between the two individual donations (Supplementary Fig. S2a,b). The heatmap on Fig. 1 shows that most population changes at the genus level differed between the two donors, subject S3 being more sensitive to genus extinctions (dark blue at bottom right of Fig. 1), and subject S2 to genus proliferation (red color at top left of Fig. 1), especially when samples were left at 37 °C for more than 24 hours.

**Table 1:** Results of the two-way ANOVA. Impact of short-term storage conditions on raw samples.

	Df	Sum of Sq	RSS	AIC	F value	Pr(>F)	
**Response variable: OTU compositional dissimilarity (Bray-Curtis) between fresh and stored raw samples**							
<none>			0.01693	−201.41182			
Time	2	0.00931	0.02624	−191.39316	3.29840	0.07219	.
Donor	1	0.00328	0.02021	−197.75319	2.32117	0.15353	
Temperature	2	0.00862	0.02555	−192.24672	3.05366	0.08472	.
Atmosphere	1	0.00003	0.01696	−203.35668	0.02070	0.88800	
Time:Donor	2	0.00054	0.01747	−204.40853	0.19110	0.82852	
Time:Temperature	4	0.01707	0.03401	−187.09967	3.02470	0.06117	.
Time:Atmosphere	2	0.00426	0.02120	−198.22825	1.51008	0.26004	
Donor:Temperature	2	0.01646	0.03340	−183.68151	5.83229	0.01700	*
Donor:Atmosphere	1	0.01097	0.02791	−187.42967	7.77362	0.01640	*
Temperature:Atmosphere	2	0.00034	0.01727	−204.78397	0.11888	0.88894	
**Response variable: distance between metabolomic profiles of fresh and stored raw samples**							
<none>			0.000814	−345.103954			
Time	2	0.002421	0.003235	−299.424696	23.798558	0.000016	***
Donor	1	0.000233	0.001046	−338.053303	4.573332	0.048244	*
Temperature	2	0.000092	0.000905	−345.261474	0.901120	0.425758	
Atmosphere	1	0.000395	0.001208	−332.868948	7.760049	0.013226	*
Time:Donor	2	0.000019	0.000833	−348.275374	0.186264	0.831828	
Time:Temperature	4	0.001432	0.002246	−316.555939	7.039911	0.001812	**
Time:Atmosphere	2	0.000172	0.000985	−342.213579	1.687543	0.216277	
Donor:Temperature	2	0.000127	0.000940	−343.901996	1.243683	0.314746	
Donor:Atmosphere	1	0.000013	0.000827	−346.531220	0.256584	0.619385	
Temperature:Atmosphere	2	0.000660	0.001474	−327.727783	6.486600	0.008649	**

Signif. codes: 0 ‘***’ 0.001 ‘**’ 0.01 ‘*’ 0.05 ‘.’ 0.1 ‘ ’ 1.

**Figure 2:**
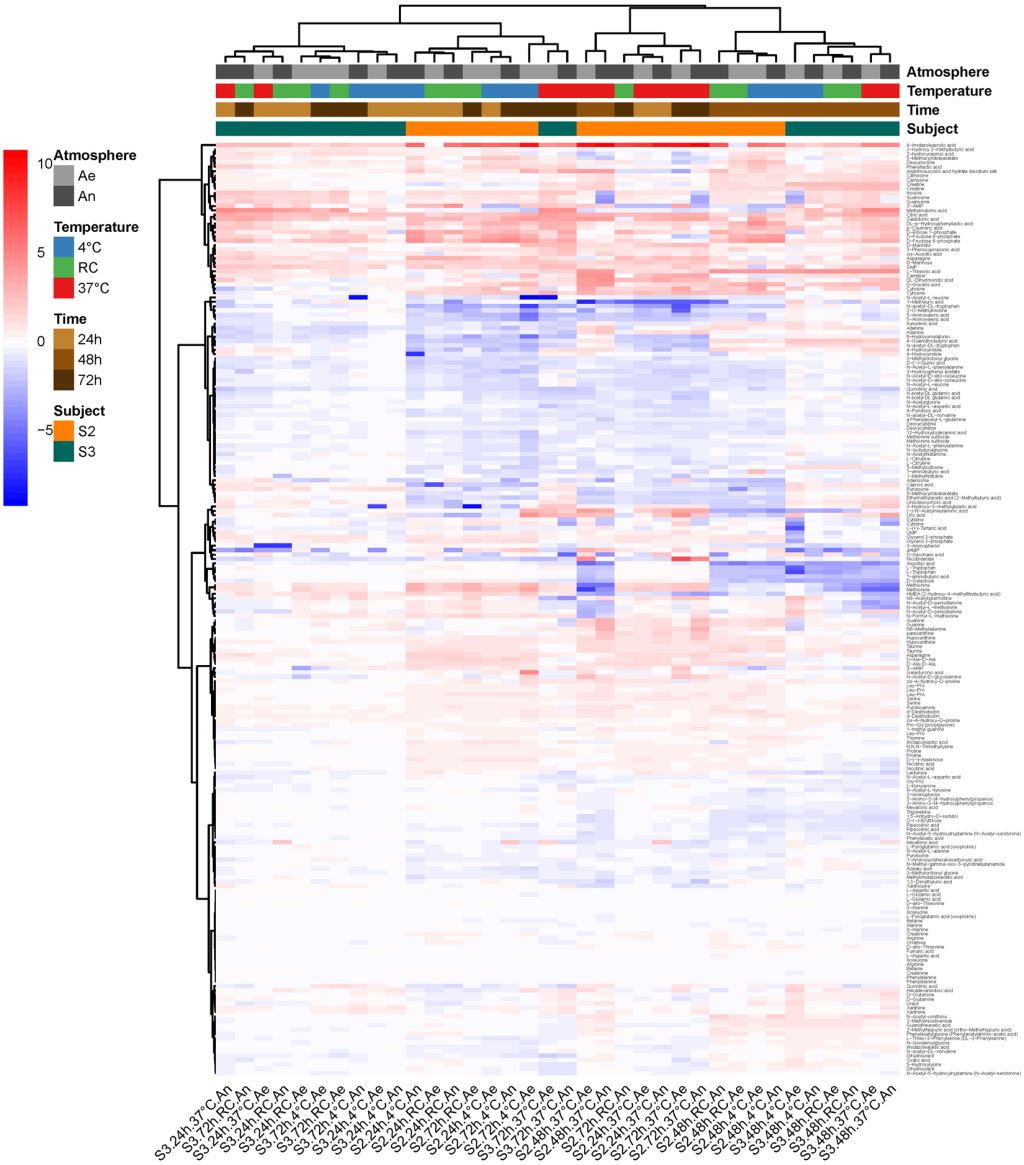
Heatmap of log2(condition/baseline) for metabolite abundance in the same raw samples as on Fig. 1. Conditions are the same as on Fig. 1. Baseline is metabolite abundance within two hours of stool collection.

For the metabolomic fingerprints, we computed Pearson correlations with baseline on Pareto reduced and quantile-normalized data. A two-way ANOVA showed a main effect of time, atmosphere and donor, as well as a two-way interaction time:temperature and temperature:atmosphere (Table 1). This means that (i) the effect of elapsed time on metabolic fingerprints of unprocessed samples differed according to temperature, with a clear shift from baseline when samples were left at 37 °C for more than 24 hours (Supplementary Fig. S2c), and (ii) the effect of high temperatures on metabolomic shift was more pronounced under anaerobiosis, indicating a persistent metabolic activity during storage, especially in samples that were left at 37 °C (Supplementary Fig. S2d). Given all the effects listed above, a clear clustering of samples did not emerge from profiles of metabolite abundance (Fig. 2). But, interestingly, changes of a given metabolite in one direction or the other was the same throughout nearly all samples, which suggests that, even though bacterial population shifts in stored samples was individual specific (Fig. 1), their activities globally resulted in the same reaction products.

### Impact of short-term storage conditions on revivification potential of raw samples

Samples of the most sensitive subject S3 were cultured in Yeast Hemin Brain Heart Infusion (YHBHI) in order to test extinction or proliferation of the bacterial communities during short-term storage. OTU composition and metabolomics fingerprint dissimilarities were computed. Overall, 752 OTUs belonging to 107 genera were quantified in cultures as compared to 964 OTUs and 113 genera in raw fecal material, proving that YHBHI broth did meet the requirements of most types of gut microorganisms and was therefore relevant to evaluate their revivification potential. The present results are in line with what could be expected from the weathering of crude samples described above: beyond a storage time of 24 hours, a temperature of 37 °C had a crucial effect on the cultures, as reflected both in microbial taxonomic composition and metabolomic fingerprints (main effect of time and significant time:temperature interaction, Table 2 and Supplementary Fig. S3).

**Table 2:** Results of the two-way ANOVA. Impact of short-term storage conditions on culturability of raw samples.

	Df	Sum of Sq	RSS	AIC	F value	Pr(>F)	
**Response variable: OTU compositional dissimilarity (Bray-Curtis) between cultures from fresh and stored samples**							
<none>			0.00698	−113.38737			
Time	2	0.17986	0.18684	−58.22207	51.52284	0.00140	**
Temperature	2	0.01139	0.01837	−99.97044	3.26328	0.14439	
Atmosphere	1	0.00909	0.01607	−100.37619	5.20963	0.08456	.
Time:Temperature	4	0.16169	0.16867	−64.06309	23.15961	0.00500	**
Time:Atmosphere	2	0.00140	0.00838	−114.10124	0.40058	0.69411	
Temperature:Atmosphere	2	0.02293	0.02991	−91.20084	6.56732	0.05450	.
**Response variable: distance between metabolomic profiles of cultures from fresh and stored samples**							
<none>			0.01099	−105.22424			
Time	2	0.21045	0.22144	−55.16391	38.30595	0.00246	**
Temperature	2	0.06706	0.07805	−73.93426	12.20653	0.01982	*
Atmosphere	1	0.00045	0.01144	−106.49642	0.16505	0.70534	
Time:Temperature	4	0.16370	0.17469	−63.43234	14.89838	0.01137	*
Time:Atmosphere	2	0.00693	0.01792	−100.42438	1.26098	0.37615	
Temperature:Atmosphere	2	0.01848	0.02946	−91.46971	3.36293	0.13908	

Signif. codes: 0 ‘***’ 0.001 ‘**’ 0.01 ‘*’ 0.05 ‘.’ 0.1 ‘ ’ 1.

**Figure 3:**
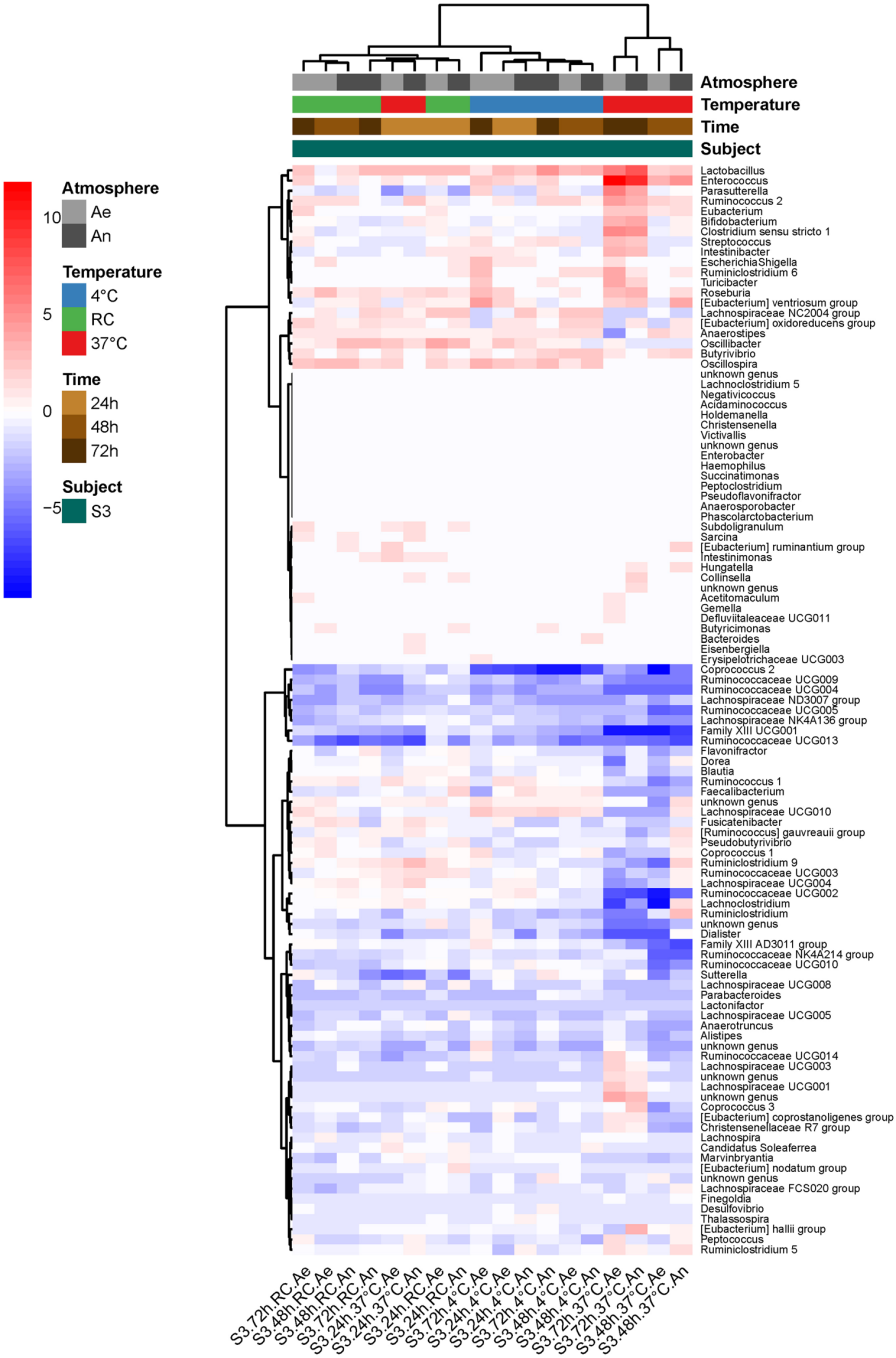
Heatmap of log2(condition/baseline) for genus abundance in cultures of a raw sample from S3 (the same as that on Figs 1 and 2). Conditions are the same as on Figs 1 and 2. Baseline is genus abundance in the culture within two hours of stool collection.

**Figure 4:**
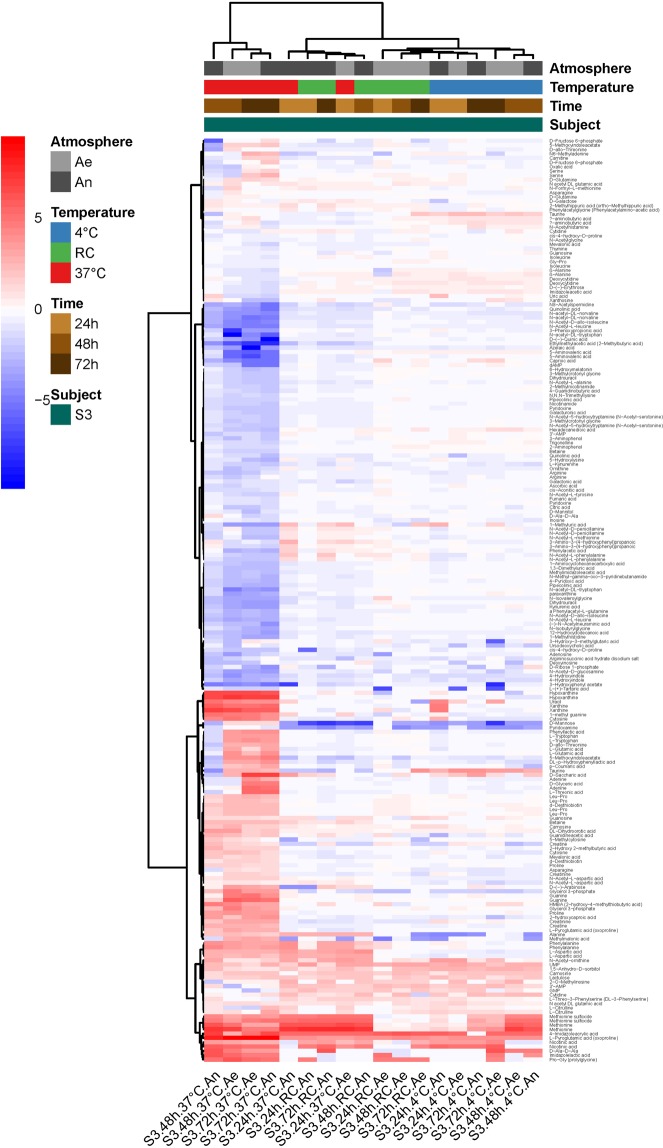
Heatmap of log2(condition/baseline) for metabolite abundance in cultures of a raw sample from S3 (the same as that on Fig. 3). Conditions are the same as on Fig. 3. Baseline is metabolite abundance in the culture within two hours of stool collection.

Figure 3 shows that most structural changes in the cultivated samples occurred in the ones that have been stored at 37 °C for more than 24 hours (cluster of four samples on the right side of the figure). Diverse genera, including *Lactobacillus*, *Enterococcus*, *Ruminococcus* 2, *Eubacterium*, and others proliferated in these conditions, while many genera from the Ruminococcaceae family, *Ruminococcus 1* or *Faecalibacterium* were in extinguishing mode. However, revivification of members from eight genera (four from Ruminococcaceae family, two from Lachnospiraceae family, *Coprococcus 2*, and Family III UCG001) was affected as early as after 24 hours of collection. The heatmap on Fig. 4 provides further evidence that tremendous metabolic changes occurred in cultures of samples that had been kept at 37 °C for more than 24 hours.

### Innovative conservative diluents and thawing conditions

We tested two maltodextrin-trehalose cocktail recipes in the ratios of 3:1 (MD) and 1:3 (TR) in normal saline, taking normal saline (NaCl) as control. Three individual donations (S1, S2 and S3, Supplementary Fig. S1) were prepared in each of the three diluents in either oxic or anoxic conditions. Each preparation was tested for its capacity to grow *in vitro*, either immediately or after two weeks of storage in a -80 °C standard freezer with thawing either rapidly in a water bath at 37 °C or gradually overnight at 4 °C. OTU composition and metabolomics fingerprint dissimilarities were computed for the 36 observations. The ANOVA in Table 3 summarizes all the effects on the OTU compositional shift between cultures before and after storage. Diluent had the greatest effect, with MD giving the overall best results, but TR could also provide excellent results for one donation (significant donor:diluent interaction, Table 3 and Supplementary Fig. S4a). The second most impacting factor was the thawing temperature, with an advantage for rapid thawing at 37 °C (Table 3 and Supplementary Fig. S4). Of note, precautions to ensure anaerobiosis during preparation of the transplants had no effect whatever the diluent, donor, or thawing temperature (Table 3).

**Table 3:** Results of the two-way ANOVA. Innovative conservative diluents and thawing conditions.

	Df	Sum of Sq	RSS	AIC	F value	Pr (>F)	
**Response variable: OTU compositional dissimilarity (Bray-Curtis) between cultures before and after storage**							
<none>			0.06273	−174.04134			
Thawing temperature	1	0.04241	0.10514	−158.47985	9.46645	0.00820	**
Donor	2	0.02550	0.08823	−166.44128	2.84623	0.09179	.
Diluent	2	0.11570	0.17843	−142.49790	12.91166	0.00066	***
Atmosphere	1	0.00335	0.06608	−174.27010	0.74867	0.40148	
Thawing temperature:Donor	2	0.00418	0.06691	−175.84762	0.46654	0.63658	
Thawing temperature:Diluent	2	0.01802	0.08074	−169.45635	2.01067	0.17076	
Thawing temperature:Atmosphere	1	0.01008	0.07281	−170.97260	2.25073	0.15576	
Donor:Diluent	4	0.22403	0.28676	−130.36641	12.50040	0.00016	***
Donor:Atmosphere	2	0.00711	0.06983	−174.39200	0.79314	0.47174	
Diluent:Atmosphere	2	0.02267	0.08539	−167.55347	2.52935	0.11541	
**Response variable: distance between metabolomic profiles of cultures before and after storage**							
<none>			4.8657E-05	−128.987438			
Thawing temperature	1	0.00026258	0.00031124	−108.71825	10.7931883	0.08148663	.
Atmosphere	1	0.00032623	0.00037488	−106.485638	13.4091863	0.06715098	.
Diluent	2	0.02703903	0.02708769	−57.122991	555.706007	0.00179628	**
Thawing temperature:Atmosphere	1	0.00013043	0.00017908	−115.350783	5.36104392	0.1465954	
Thawing temperature:Diluent	2	0.00018688	0.00023554	−114.06238	3.84083958	0.20657574	
Atmosphere:Diluent	2	0.00561106	0.00565972	−75.9114546	115.318485	0.00859709	**

Signif. codes: 0 ‘***’ 0.001 ‘**’ 0.01 ‘*’ 0.05 ‘.’ 0.1 ‘ ’ 1.

**Figure 5:**
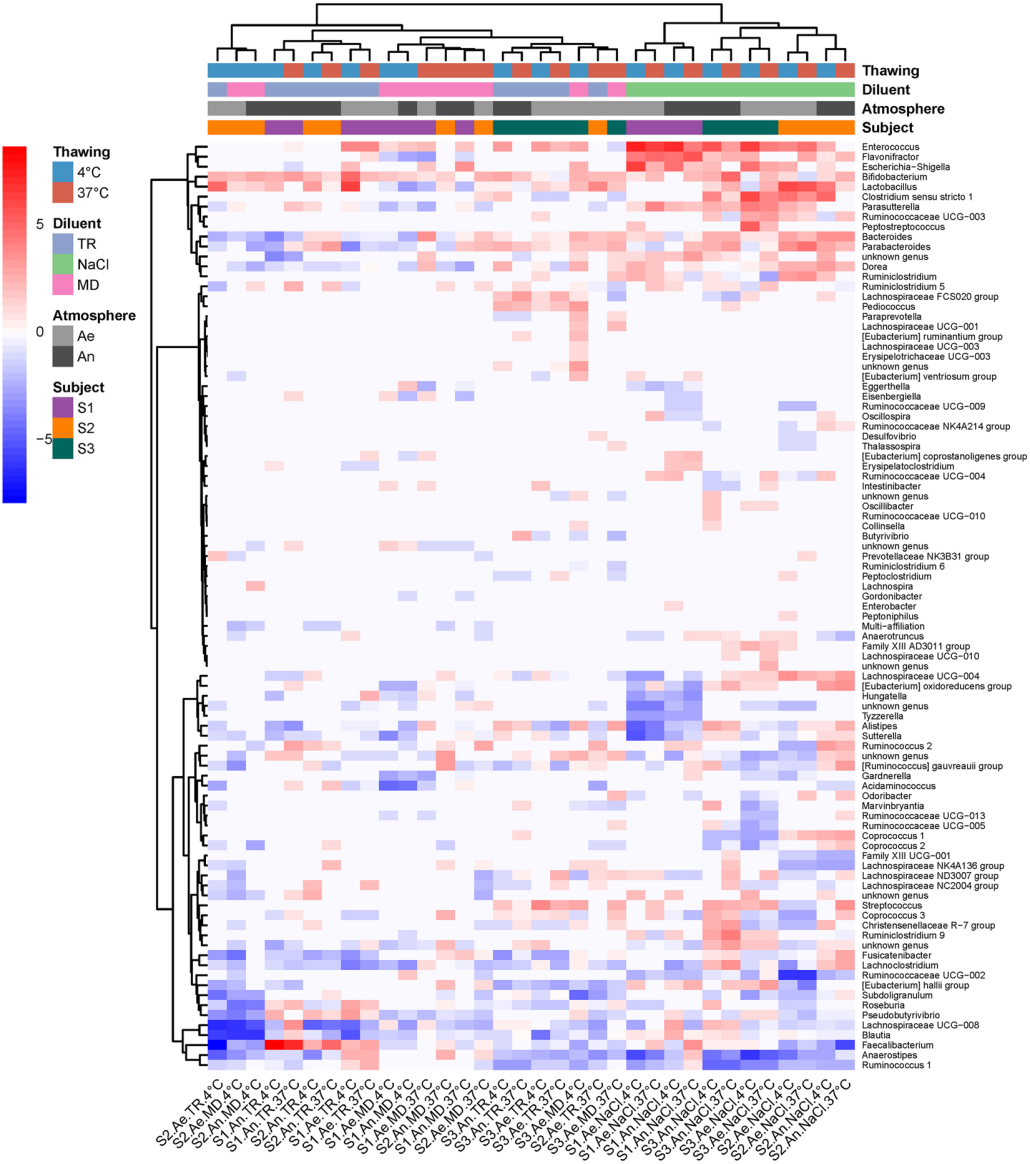
Heatmap of log2(condition/baseline) for genus abundance in cultures of fecal transplants (from three individuals S1, S2 and S3) that have been frozen at -80 °C for two weeks. Conditions are combinations of conditioning atmosphere (An and Ae for anaerobiosis and aerobiosis, respectively), diluent (NaCl, MD or TR), and thawing protocol (5 min at 37 °C in a water bath or overnight at 4 °C). Baseline is genus abundance in the transplants before freezing.

The heatmap on Fig. 5 clearly shows that, for all three donations, revivification of the culturable bacterial community was most affected after freezing in NaCl (cluster of samples on the right side of Fig. 5), with proliferation of a group of three genera, corresponding to *Enterococcus*, *Escherichia-Shigella*, and *Flavonifractor*, and extinction of the genera *Faecalibacterium, Anaerostipes and Ruminococcus 1*. Figure 5 also confirms that cryoconservation in MD with rapid thawing at 37 °C was the most favorable condition, giving post-freezing cultures that most resembled pre-freezing cultures, with the lowest numbers of over-proliferations and extinctions (lighter- colored columns of the figure). In particular, members from the Ruminococcaceae and Lachnospiraceae families that extinguished in NaCl transplants were well preserved, and proliferation of Enterobacteriaceae was not observed. Slow defrosting at 4 °C led to poorer results, notably extinction of *Blautia* and *Faecalibaterium*. As highlighted hereinbefore by ANOVA, TR transplant defrosted at 37 °C also gave excellent results for the S3 donation.

**Figure 6:**
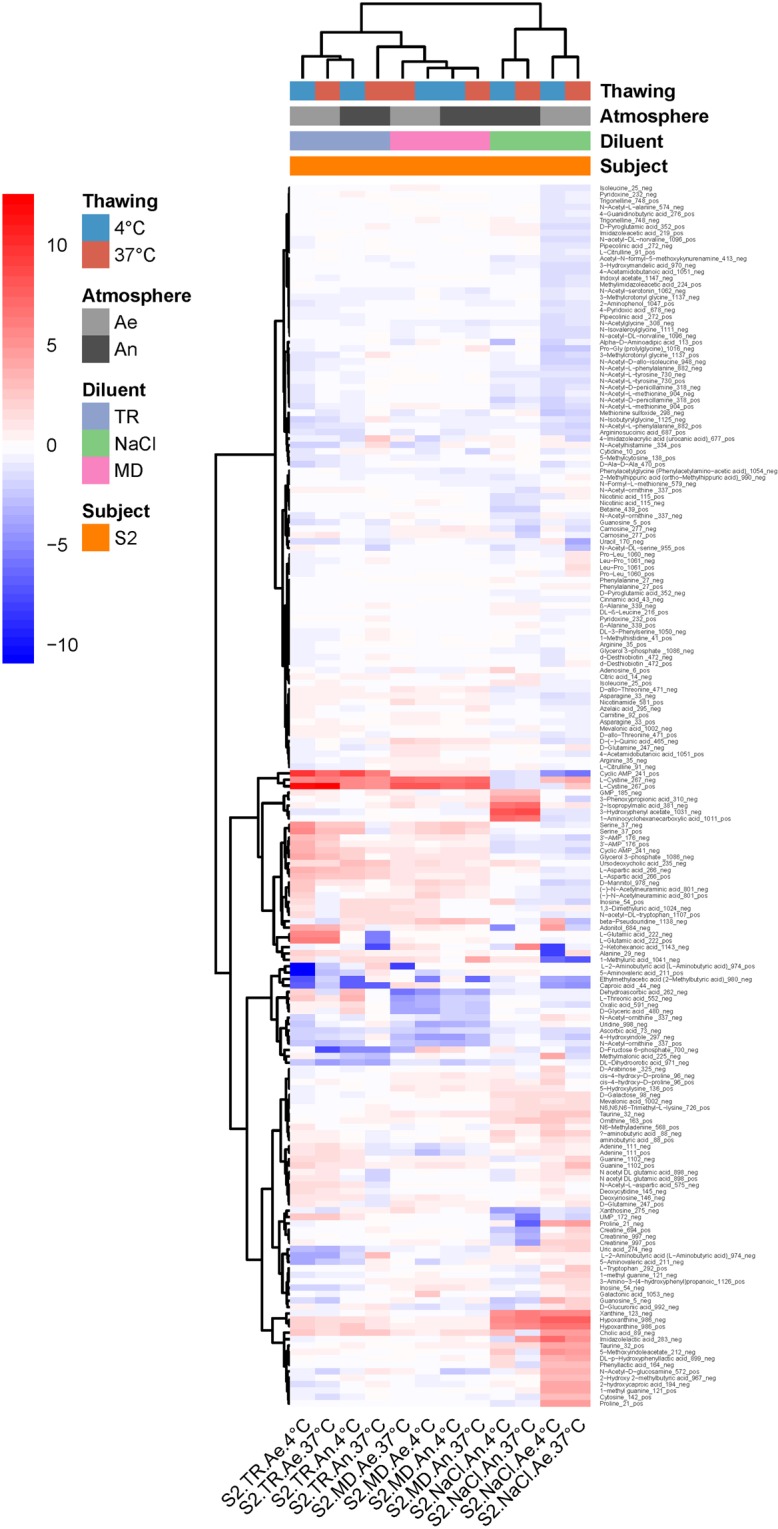
Heatmap of log2(condition/baseline) for metabolite abundance in cultures of fecal transplants (from S2, the same as on Fig. 5) that have been frozen at -80 °C for two weeks. Conditions are the same as on Fig. 5. Baseline is metabolite abundance in the transplants before freezing.

Consistent with all above results, we found that metabolomic fingerprints of cultures from defrosted suspensions were mainly influenced by the diluent (Table 3), with a clear advantage of MD and TR over NaCl in maintaining a metabolic activity close to that observed in cultures of freshly prepared transplants (Supplementary Fig. S4c). In addition, we found a significant diluent:atmosphere interaction (Table 3), with a small advantage for anaerobiosis when transplants were prepared in MD and TR (Supplementary Fig. S4d). Figure 6 highlights the metabolites that mainly differed between cultures of fresh and defrosted transplants, and how much the figures differed between cryoconservation in NaCl on the one hand, and MD or TR on the other.

### Viability control of fecal transplants by flow-cytometry

**Figure 7:**
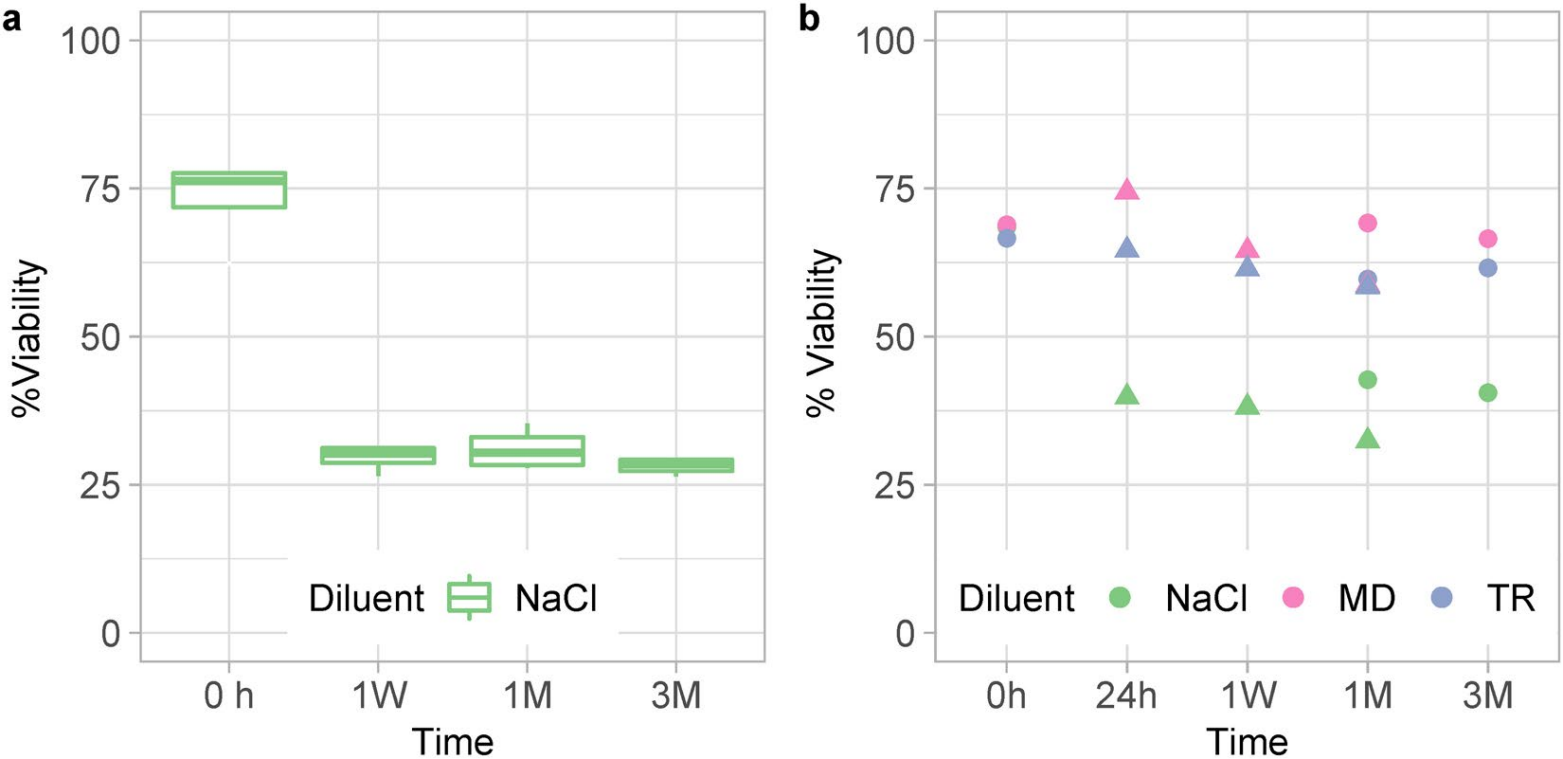
Percent live bacteria in transplants prepared in NaCl (**a**, four donations from subjects S2, S3, S4 and S5), or in either NaCl, or MD, or TR (**b**, two different donations from subjects S2 -round dots- and S4 -triangular dots-). Transplants were frozen by fractions in a -80 °C standard freezer, and then rapidly thawed at defined times (24h: 24 hours, 1W: 1 week, 1M: 1 month, 3M: 3 months) for live/dead tests by flow cytometry. Viabilities at 0 hour are those measured before freezing.

We implemented a live/dead test by flow cytometry for routine analysis of bacterial viability in fecal transplants at different steps of the process. All preparations were conditioned under anaerobiosis and were frozen by fractions in a -80 °C standard freezer. At time intervals up to 3 months, the fractions were rapidly thawed at 37 °C for live/dead test. In a first set of assays with transplants of four subjects in NaCl, percent live bacteria was at around 75% in freshly prepared suspensions, and dropped to about 30% (p = 2.8 × 10^−8^) after just one week of cryoconservation, without further loss within three months of freezing (Fig. 7a). In a second set of experiments with transplants of two subjects in either NaCl, or MD, or TR as diluents, MD and TR had a clear cryoprotective effect compared to NaCl (p = 4.3 × 10^−7^), retaining more than 60% viability over a three-month conservation period, a percentage only just below freshly prepared transplants (Fig. 7b). Interestingly, bacterial death essentially occurred within 24 hours of cryoconservation in all preparations (Fig. 7b). This means that the freezing-thawing cycle rather than duration of cryoconservation in a -80 °C standard freezer, here limited to three months, was the main cause for the loss of bacterial viability.

### Viability of freeze-dried transplants

Finally, we tested the survival of fecal transplants in their lyophilized state, a formulation that can be attractive for a more convenient storage and/or encapsulation for oral administration. Briefly, suspensions prepared in the three reduced diluents from three different donors, were freeze-dried within the week. After ten months of storage at room temperature within sealed air and moisture-tight aluminum/plastic compound bags, they were reconstituted to their original volume with reduced ultrapure water. Viability in the reconstituted samples was still nearly 30% in MD and 25% in TR, while it dropped to about 14% in NaCl (main effect of the diluent, p = 0.017, Supplementary Fig. S5). Interestingly, there was no main effect of the donor (p = 0.066).

### Viability of residue-free microbiota

**Figure 8:**
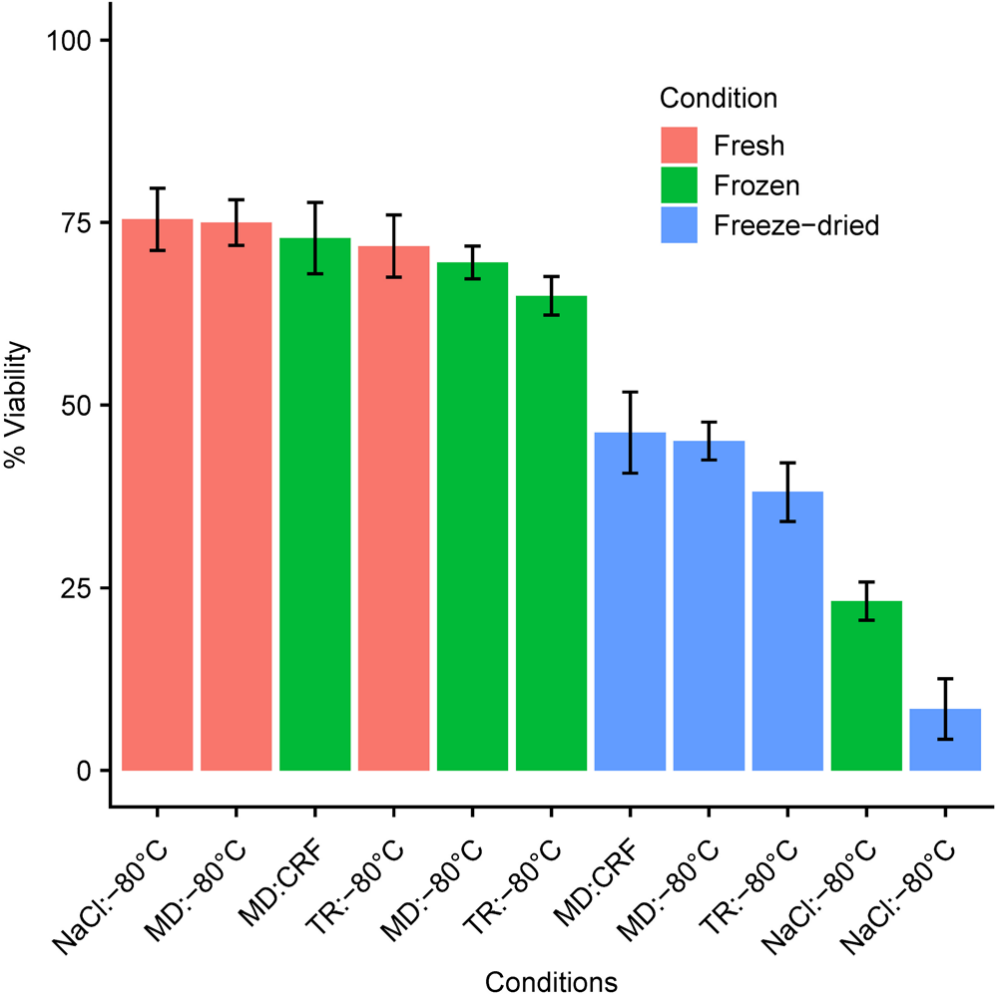
Percent live bacteria in fresh, frozen or further freeze-dried purified microbiota in either NaCl, or MD, or TR. Samples are ranked in descending order of viability (live/dead test by flow cytometry). Statistics are detailed in Supplementary Table S1. The black bars are 95% confidence intervals.

To progress towards oral administration of most appropriate encapsulated forms of lyophilized transplants, we extracted either by gradient or differential centrifugation, the microbiota from fourteen stool samples provided by four individuals (Supplementary Fig. S1). Both purification methods yielded an average of 30% bacterial cells with a relative OTU composition close to the original samples^15^. Each of the fourteen purified microbiota pellet was divided into three equal parts that were resuspended in a minimal volume of either NaCl, or MD, or TR, each of them being frozen in aliquots in a -80 °C standard freezer (uncontrolled cooling rate, average global value of 0.8 °C/min), or at 1 °C/min to −100 °C in a controlled-rate freezer (CRF). Part of the constituted sample stock was further lyophilized within the week and then stored at room temperature within sealed air and moisture-tight aluminum/plastic compound bags or sealed amber glass vials for up to three months (Supplementary Fig. S6). As shown on Supplementary Fig. S7, loss of viability in purified microbiota, either simply frozen or freeze-dried, was observed from the very first measurements i.e. within one week or even 24 hours of conservation, and remained stable thereafter. We therefore conducted an ANOVA where all observation times after freezing or freeze-drying (one week, one month, two months or three months) were grouped together. On Fig. 8 and in Supplementary Table S1, samples were ranked in descending order of viability, which was as great in microbiota frozen in MD or TR as in freshly prepared microbiota, reduced but still high in samples freeze-dried in MD or TR, low in samples frozen in NaCl, and the lowest in samples freeze-dried in NaCl. Importantly, subject effects were smaller than treatment effects (Supplementary Table S1), indicating that these results are likely to be reproducible for any donor.

### A rational and informed process for collecting, conditioning, storing and reconditioning viable fecal transplants

The process was deduced from the results obtained above and was definitely set as Standard Operating Procedures (SOPs) and directly transferred to pharmaceutical pilot scale assays. The early stage of the process involves self-collection of stools in dedicated devices (Figure S8a) that are transferred within less than 72 hours to the pharmaceutical laboratory for conditioning. If, for logistical reasons, waiting time would be greater than 24 hours, then it will be crucial not to expose samples to temperatures above 20 °C. In that respect, refrigeration at 4 °C for short-term storage and shipment can be a preferred solution. The diluent is prepared at a controlled temperature of 40 ± 5 °C to achieve dissolution of the ingredients that are all pharmaceutical grade and gluten-free. Then it is degassed for 3 h before addition of ascorbic acid and cysteine as reducing agents at a controlled pH, finally online filtered on a 0.22 µm polyethersulfone (PES) membrane, and distributed in O_2_-proof 500-mL bags. The bags are stored at a controlled temperature of 20 ± 2 °C for a maximum of 60 days, and used for direct online transfer to the stool collection device, at a dosage of 4 mL per g of stool. Stool dilution and filtration are preformed within the device itself. The obtained inoculum is directly transferred to 150-ml bags dedicated for FMT use (Supplementary Fig. S8b). Composed of USP Class VI materials including an O_2_-barrier film, the bags display specific connections to avoid miss-use. They are immediately stored at -80 °C. Upon administration, the inoculum-containing bag is rapidly thawed at 37 °C and immediately connected to the rectal probe via a special tubing. After air removal, the probe is installed, and instillation can begin by simple gravity, taking about 15 minutes for complete administration. No discomfort has been reported by the patients^23^. Over a period of one year, we performed a hundred viability tests on a reference sample to certify the measurement of transplant viability by flow cytometry. This resulted in a SOP for routine analysis of transplant viability. Briefly, non-degassed NaCl is used for serial dilutions, and all manipulations are performed under a laminar flow hood. Several dilutions of the same sample were tested to assess the most relevant ones on the equipment (Accuri C6), and 10^−3^ and 10^−4^ were selected for routine analyses. Staining protocol is as described in the section ‘Materials and Methods’ using the BacLight kit, and a standardized protocol is used for data collection and interpretation. On a series of fourteen pre-clinical transplants prepared in MD from seven individual donations (Supplementary Fig. S1), integrating all stages of the pharmaceutical stool collection and transplant production process, we recorded a mean loss of only 9.8% of bacterial cell viability after three months of storage in a -80 °C standard freezer, compared to the corresponding freshly prepared transplants (Supplementary Fig. S9).

## Discussion

In the present paper, we conducted a systematic study that provides basic but hitherto not available data to help ensure standardized preparations and conservation of ready-to-use transplants. The results meet the requirement for a standardized process for collecting, conditioning, storing and reconditioning fecal suspensions or purified microbiota intended for FMT. The early phase of the process entails self-collection of stools. In the development study, we followed our SOP where feces are directly collected in a Stomacher® 400 bag, which is placed in a catalyst-containing box^24^. Then, in a pilot pharmaceutical study, we developed and patented a new dedicated easy-to-use device^25^. It enables anaerobic and hygienic self-collection of stool samples, and subsequent conditioning by the pharmaceutical unit (introduction of the diluent, homogenization, filtration, and direct transfer to the perfusion bag) without opening the device, thus preventing any contact of the patient or the manipulator with the sample, and any exposure of the sample to the environment.

Time that may elapse between collection and conditioning of stools, as well as temperature and atmosphere conditions have received little attention if any at all and are not comprehensively informed in most available reports, while these factors are well-known to affect integrity of other tissues^26^ and food^27^. Based on ANOVA of microbial shift in OTUs abundances within stored compared to fresh samples, we found that sensitivity to short-term storage conditions (temperature and atmosphere) differed between individual donations. We confirmed that extinctions and proliferations of genus were specific to each individual, and predominantly occurred in stools left at 37 °C for more than 24 hours. Consistent with these compositional changes, we found that for both donations, metabolomic fingerprints of stored samples departed more and more from baseline as time increased, the deviation being much more pronounced when samples were kept at 37 °C. We further investigated the revivification potential of one individual sample that had been stored up to 72 hours under different temperature and atmosphere conditions. When compared to immediate culture, taxonomic changes at the OTU level and metabolomic shifts in the delayed cultures were those expected from the weathering previously observed on the crude sample, namely a non-significant impact of storage temperature if waiting time did not exceed 24 hours, but a deleterious effect of temperature above 20 ± 2 °C, beyond that storage period. However, when looking at the genus level, we could identify a group of eight genera among more than a hundred, whose revivification in culture was affected as early as the first 24 hours of storage. They were essentially from the families Ruminococcaceae and Lachnospiraceae.

Therefore, best conditions for storing fecal material dedicated to processing for FMT were definitely set as SOPs and directly transferred to pharmaceutical pilot scale assays. The early process involves self-collection of stools in dedicated devices that are transferred within less than 72 hours to the pharmaceutical laboratory for conditioning. If, for logistical reasons, waiting time would be greater than 24 hours, then it will be crucial not to expose samples to temperatures above 20 °C. In that respect, refrigeration at 4 °C for short-term storage and shipment can be a preferred solution. Of note, oxygen-free atmosphere can be advantageous for some individual donations. For this reason, the above-mentioned device was equipped with a port allowing to expel air above the stool sample, and we could verify that the partial pressure of oxygen (pO2) in the inoculum was in fact very low (<0.1%).

Conservation of transplants is essential to give rapid access to fecal material whose safety has been tested upstream in an allogenic context, and is an inherent and necessary part of the protocol in autologous FMT. The currently recommended protocol advocates the use of glycerol at a final concentration of 10% for conservation of frozen fecal suspensions^20^, but glycerol at 10% is not suitable for downstream lyophilisation as it leads to an insufficiently dry and a sticky product^28^. In this work, we emphasized the selection of cryoprotective agents that will allow lyophilisation as an optimal means of progressing towards oral administration of encapsulated forms. We selected the two well-known extracellular cryoprotectants maltodextrin and trehalose since they help in increasing the medium viscosity, thus limiting ice crystallization and osmotic imbalance that occurs during freezing^29,30^. They differ from intracellular cryoprotectants such as dimethyl sulfoxide and glycerol, which can freely penetrate cell membranes and then compromise original biological and metabolic characteristics at revivification.

We tested two maltodextrin-trehalose cocktail recipes in the ratios of 3:1 (MD) and 1:3 (TR) in normal saline, taking normal saline as control. Moreover, anti-oxidative additives, namely ascorbic acid and cysteine, were extemporaneously added in all anaerobic preparations. For three individual donations, we showed that cultures of transplants conditioned in MD for storage in a -80 °C standard freezer, and then later rapidly thawed at 37 °C, most resembled the fresh cultures, based on OTU abundances and metabolomic profilings. Importantly, when looking at the genus level, we found an overproliferation of members of the genera *Enterococcus*, *Escherichia-Shigella* and *Flavonifractor*, and an extinction of *Faecalibacterium, Anaerostipes* and *Ruminococcus 1* in the post-freezing cultures of all NaCl transplants. Thawing of sugar-protected transplants at 37 °C better conserved viability of a beneficial population core, including members belonging to the families Ruminococcaceae and Lachnospiraceae of the Firmicutes phylum, and prevented proliferation of pro-inflammatory Proteobacteria. As dominant butyrate producers are preserved, the preparations should allow a better recovery of this function with expected benefits in terms of restoration of immune homeostasis. This may not be essential for the treatment of rCDI, but critical for immune-related clinical indications for which maintaining a high integrity of functional groups in the preparation may be critical and most favorable to obtain a consistent improvement of the patient’s condition. Fairly surprisingly but in line with our previous results which downplayed the effect of atmosphere on integrity of waiting samples, we found that oxygen exclusion during transplant manufacturing was not an essential prerequisite for ensuring survival of most bacterial populations, at least in rapidly conditioned stool suspensions. The precautions highlighted would hence allow to benefit from stools inherent reducing capacity to protect most oxygen-sensitive bacterial taxa of the Firmicutes phylum.

In a series of live-dead tests using flow cytometry, we could confirm the true efficacy of the newly developed diluents for retaining a high viability of bacterial populations in frozen-thawed transplants, either in their crude or purified form, with a small edge of MD over TR in both cases. This is in line with conclusions previously drawn from our culturomics experiments. Indeed, although it might be difficult, if not impossible, to directly correlate both approaches, each one leads to the conclusion that revivifiable members were least affected in the new diluents. That flow cytometry can advantageously replace traditional microbiological methods was proved in more simple systems^31,32^. Since viability in the defrosted, raw and purified transplants was nearly as high or even indistinguishable from that in the fresh preparations, we can reasonably propose the new diluents as an alternative solution for viable and ready-to-use transplants that circumvents the use of glycerol. Of note, when death occurred, typically in samples frozen in normal saline, this was the result of the freezing-thawing cycle, and did not aggravate as the frozen period increased from 24 hours up to the 3-month limit of our observation time. The freezing methods applied, involving either uncontrolled or controlled cooling rates, did not exhibit different impact on viability, possibly because global average cooling rates (0.8 and 1 °C/min, respectively) was similar in both cases, or simply because viability was already maximum with uncontrolled cooling rates.

To progress in the near future towards oral administration of encapsulated forms of transplants, we tested the viability of freeze-dried transplants, either in their crude or purified form, where bacterial communities were extracted from the raw fecal material before being freeze-dried. Interestingly, the benefit of the newly developed diluents over NaCl was more important for the purified form (mean viabilities of 45, 38 and 8% for MD, TR and NaCL, respectively, Fig. 8) than for the crude form (mean viabilities of 30, 25 and 14% for MD, TR and NaCl, respectively Supp. Fig. S5). This might be the result of two independent effects: (i) a physical protective effect of the fecal matrix during freeze-drying in NaCl (as viability was higher in NaCl crude lyophilisates than in NaCl purified lyophilisates), (ii) presence of bioactive compounds toxic to bacterial cells in crude lyophilisates (as viability in MD and TR crude lyophilisates was lower than in MD and TR purified lyophilisates). Therefore, although lyophilisation was more detrimental than simple cryoconservation, leading to undamaged cell percentages that fell within the range of those reported by others^33,34^ for diverse pure bacterial strains in sugar-based solutions, MD and TR efficiently maintained viability during freeze-drying. Another group^22^ recently reported much higher viabilities of encapsulated 5% trehalose freeze-dried transplants, indistinguishable from the initial fresh material. This might be explained by either a remarkably improved lyophilization protocol, or a much lower sugar content in the protective solution, or simply different appreciation of undamaged cells, by microscopy in their study *versus* flow cytometry in ours and others^33,34^. This bench to bedside study further demonstrated the successful resolution of rCDI in 88% of patients who received the capsule treatment. Therefore, the upgraded version of freeze-dried transplants in purified form might well be introduced in a near future, if their clinical effectiveness is demonstrated. Automation of the purification process would not be a problem in itself since differential centrifugation or even bacterial fraction recovery from gradients under camera control is already robotized for many other biological and pharmaceutical applications. Crossflow filtration is an additional affordable option to obtain pure bacterial fractions^35^.

In conclusion, transplants prepared in maltodextrin-trehalose solutions, stored in a -80 °C standard freezer and then rapidly thawed at 37 °C, retained the best revivification potential based on a series of *in vitro* assays (culturomics and flow cytometry). Maltodextrin-trehalose containing cryoprotectants were also efficient in preserving viability of lyophilized transplants, either in their crude or purified form, an option that can be attractive for fecal transplant biobanking and oral formulation. This is a first step before preclinical assays of engraftment of the proposed preparations in an animal model, and ultimately clinical assays dedicated to the reconstruction of a functional microbiota.

## Methods

### Volunteers

A total of twelve healthy volunteers with neither symptoms nor a family history of gastrointestinal disorder and with no use of antibiotics within the preceding two months participated in the study that was approved by the ethics committee CPP of Ile de France 1 under number 13642 (Supplementary Fig. S1). Informed consent to the protocol was obtained from all subjects, which were asked to provide fresh stool samples collected at home in Stomacher® 400 bags (Seward Medical) which were left open in 1 L plastic containers (216-0417 from VWR) under anaerobic conditions using a catalyst (Anaerocult, Merck, Darmstadt, Germany) according to the International Human Microbiome Standards^24^. All methods in this study were performed in accordance with the relevant guidelines and regulations.

### Chemicals

Chemicals used for diluent manufacturing were maltodextrin from Roquette, trehalose 90210 from Fluka, NaCl 27810-195 from VWR, L-Cysteine hydrochloride monohydrate C7880 and Sodium L-ascorbate A7631 from Sigma. In addition, we used Bacto™ Brain Heart Infusion 237500 and Bacto™ Yeast Extract 212750 from BD, and Hemin H9039, D-(+) Cellobiose 22150 and D-(+)-Maltose monohydrate M5885 from Sigma, to prepare the culture medium. We used OptiPrep™ 1114542 from Proteogenix, and pharma grade Hepes PHG0001 from Sigma for microbiota purification by gradient.

### Stool diluents

Two maltodextrin-trehalose cocktail recipes in the weight ratios of 3:1 or 1:3 in normal saline were tested and compared to normal saline. They are referred as to ‘MD’, ‘TR’ and ‘NaCl’. Maltodextrin and trehalose were dissolved in normal saline (9.0 g/L NaCl) at 40 °C, to respective final concentrations of 18.75% and 6.25% (w/v) for MD, or 6.25% and 18.75% (w/v) for TR. The diluents were then filter-sterilized on PES membrane 0.22 µm and degassed in a 90 °C water bath for 30 min. When used under anaerobiosis, diluents were allowed to stand at ambient temperature (20 ± 2 °C) of the working anaerobic Freter chamber for at least 48 hours before use. Any adaptation made for pharmaceutical preparations are described in the discussion section.

### Impact of short-term storage conditions

Each stool sample was transferred to the anaerobic Freter chamber (90% N_2_, 5% H_2_ and 5% CO_2_) within 2 hours after collection. It was homogenized for 5 min by manual kneading through its collection bag, and divided into fractions of 5–10 g in Stomacher® 400 bags (Seward Medical), which were left 24 to 72 hours in the defined atmosphere-temperature conditions. Diversity and metabolic activity were assessed in raw and cultured samples at baseline and different observation times, based on microbial taxonomic (16S rRNA) and metabolomic profilings.

### Testing of innovative conservative diluents

For each donation, eight fractions of 15–20 g were transferred into eight Stomacher® Filter Bags (Seward BA6041/8TR, or VWR 432–3119, 0.5 mm holes) within the anaerobic chamber. From this point, conditioning was under anaerobic conditions for four bags that remained within the anaerobic chamber (identified An), while the four remaining bags were transferred out of the chamber and handled under aerobiosis in a regular hood (identified Ae). Anaerobic and aerobic conditionings were implemented within the same three filter-sterilized pre-reduced diluents – referred as MD, TR and NaCl – by two well-trained two member teams, who operated in parallel. Four ml of diluent were added per g of stool. Dilutions under anaerobiosis were further supplemented with two reducing agents, sodium L-ascorbate (Sigma A7631) and L-cysteine hydrochloride monohydrate to a final concentration of 0.5% (w/v) and 0.05% (w/v), respectively (stock solutions of L-ascorbate 50% and L-cysteine 5% were prepared within the anaerobic chamber, using reduced double-distilled water). Five-min hand mixing through the filter bags (closed by clips, VWR 432-3116) ensured both homogenization and filtration. Then, 20 mL of each of the six filtrates (identified NaCl-An, MD-An, TR-An, NaCl-Ae, MD-Ae, and TR-Ae) was pipetted into two CryoMACS® Freezing Bags 50 (Miltenyi Biotec SAS 200-074-400), giving a total of twelve bags per donor, which were stored in a -80 °C standard freezer, positioned flat on the bottom floor of the freezer. Thawing was carried out after two weeks of storage, either overnight at 4 °C or 5 min in a water-bath at 37 °C. Diversity and metabolic activity were assessed in raw and cultured transplants, before and after freezing, based on microbial taxonomic (16S rRNA) and metabolomic profiling.

### Revivification potential

We probed the revivification potential of culturable bacterial communities in the anaerobic, highly nutritive and versatile liquid infusion medium YHBHI (yeast-hemin-brain heart infusion), known to meet the requirements of many types of gut microorganisms^36^. For stool samples, aliquots of 0.4 g were five-time diluted by adding 1.6 mL of YHBHI. Aliquots of 0.5 mL of these suspensions were used to inoculate three screw cap Kimble™ Kimax™ culture tubes containing 9.5 mL YHBHI. For transplants (either freshly prepared or after thawing), 0.5 mL of each formula to be tested, served for the inoculation of three Kimax culture tubes containing 9.5 mL YHBHI enriched with Sodium L-ascorbate, L-Cysteine hydrochloride monohydrate, Cellobiose D-(+), and Maltose monohydrate (Supplementary Methods). All cultures were incubated for 48 hours at 37 °C within the chamber. At harvesting, triplicate cultures were pooled and centrifuged at 5000 × *g*, 4 °C for 30 min; the wet pellets were kept for microbial taxonomic (16S rRNA) profiling, while supernatants were further ultra-centrifuged (220,000 × *g*, 4 °C for 1 h) for metabolomic profiling.

### RNA extraction and microbial taxonomic (16S rRNA) profiles

Total RNA was extracted from stool aliquots (150–200 mg), from fecal suspension pellets, or from bacterial culture pellets using Diethylpyrocarbonate (DEPC) water and RNAse-free reagents. A chemical lysis step with phenol chloroform was combined with a Fast Prep mechanical lysis before using the High Pure RNA Isolation kit (Roche 11 828 665 001) following a previously described protocol adapted to the recovery of RNA from strains originating from a complex environment^37^.

Microbiota composition was assessed by 454 pyrosequencing (innovative conservative diluent section) or Miseq sequencing (impact of short term storage condition section) targeting the V3-V4 region of the bacterial 16S rRNA gene (V3fwd: 5′TACGGRAGGCAGCAG3′, V4rev: 5′GGACTACCAGGGTATCTAAT3′). Sequencing was subcontracted to the company GenoScreen. Bioinformatic analyses were performed with FROGS pipeline^38^ (detailed in Supplementary Methods).

### Metabolomic profiles

Metabolomic profiling was performed by the company Profilomic (92100 Boulogne Billancourt, France). Briefly, 50 µL of inoculum or culture centrifugation supernatant were deproteinized with four volumes of methanol. The deproteinized sample was dried, supplemented with the internal standard, and centrifuged. Analysis was carried out on 60 µL of supernatant diluted in acetonitrile. Chromatographic separation was performed over a 30 min mobile phase gradient, on a Sequant ZIC-pHILIC column (Merck, Darmstadt,Germany). Detection was achieved using a Q-Exactive mass spectrometer (Thermo Fischer Scientific) fitted with an electrospray source and operated in positive and negative ion modes. Spectra were interpreted by referencing to an internal library of more than 1000 compounds. In our study, 100–150 unique metabolites were identified and used for the analysis of distances between samples.

### Preparation of residue-free transplants

Extraction of microbial communities from the fecal matrix either by gradient or differential centrifugation is detailed in Supplementary Methods.

### Freezing

Freezing was performed by two methods: (i) placing samples in a -80 °C standard freezer (global average cooling rate of 0.8 °C/min between -10 °C and -50 °C, but not constant rate of cooling with 2 °C/min up to -30 °C, about 0.5 °C/min from -30 °C to -50 °C, and <0.4 °C up to the lowest temperature); (ii) applying controlled freezing at 1 °C/min in a controlled-rate freezer (CRF) (VIA Feezer™, Asymptote Ltd., Cambridge, UK) up to -100 °C.

### Lyophilisation

Lyophilisation was performed by the company Synerlab-Lyofal (13300 Salon-de-Provence, France), at -40 °C under deep vacuum for 48 hours. Flasks and bags were sealed under vacuum.

### Live/dead viability by flow cytometry

Viability was measured using the LIVE/DEAD® BacLight Bacterial Viability Kit (L7012 Molecular Probes) based on membrane integrity (synonymously used here for viability). Serial dilutions of the samples (either freshly prepared or defrosted fecal suspensions, or reconstituted freeze-dried samples) were rapidly carried out in sterile normal saline, down to ~10^6^ bacteria / mL, and 1 mL of the final dilution was immediately stained with 1.5 µL of a 50:50 (v/v) mix of components A (SYTO 9) and B (Propidium iodide) purchased in the BacLight kit. Each dilution step and staining was followed by 6 sec high speed vortexing. Stained samples were incubated for 15 min in the dark at room temperature. Data were acquired at 488 nm (blue laser), within 30 min following staining, on a CyFlow Space Partec-Sysmex flow cytometer. Gating of live and dead populations is detailed in Supplementary Methods.

### Statistics

For 16S rRNA, analyses were performed in the R environment with the library phyloseq. Samples were rarefied to the same sequencing depth for each experiment to 529 sequences for 454 sequencing (test of innovative diluents) and 9007 sequences for MiSeq sequencing (test of short-term storage conditions). We then computed ecological distances with Bray-Curtis dissimilarity measures. By the same way, we computed the distances between metabolomics profiles based on Pearson correlations of Pareto reduced and quantile-normalized data. We performed two-way ANOVAs in the R environment using the lm and drop1 functions in order to identify conditions that impacted the ecosystem. Normality and homogeneity of the variance were checked. To identify communities and metabolites that change in the different conditions, we computed the log2 ratio (condition/baseline), using as baseline: samples at the condition “time = 0 h” for short term storage, and at the condition “before freezing” for innovative diluents. Heatmaps of log2 ratio values were drown with the R pheatmap library (parameters: distance = euclidean, clustering method = Ward.D). For flow cytometry, we performed an ANOVA with subject and conditions effects using lm and drop1 functions, and computed 95% confidence intervals for each condition. All figures were drawn using the R library ggplot2.

## Supplementary information

Supplementary Information

## Data Availability

The nucleotide sequencing data that support the findings of this study are available in the European Nucleotide Archive repository, with accession numbers PRJEB30319 and PRJEB30320 for short-term storage, and conservative diluents, respectively.
