# Supplementary material for: A Guide for Ex Vivo Handling and Storage of Stool Samples Intended for Fecal Microbiota Transplantation
Source: Sci Rep. 2019 Jun 20;9:8897. doi: 10.1038/s41598-019-45173-4 (PMC6586871; doi:10.1038/s41598-019-45173-4)
Supplement: Supplementary file 1 — Supplementary Information [file 41598_2019_45173_MOESM1_ESM.pdf]

A Guide for *Ex Vivo* Handling and Storage of Stool Samples Intended for Fecal Microbiota Transplantation

Sebastian D. Burz[1,4], Anne-Laure Abraham[2], Fernanda Fonseca[3], Olivier David[2], Audrey Chapron[1], Fabienne Béguet-Crespel[1], Stéphanie Cénard[3], Karine Le Roux[1], Orlane Patrascu[1], Florence Levenez[4], Carole Schwintner[5], Hervé M. Blottière[1,4], Christel Béra-Maillet[1], Patricia Lepage[1], Joël Doré[1,4] & Catherine Juste[1,*]

[1]Micalis Institute, INRA, AgroParisTech, Université Paris-Saclay, 78350, Jouy-en-Josas, France. [2]MaIAGE, INRA, Université Paris-Saclay, 78350, Jouy-en-Josas, France.

[3]GMPA, INRA, AgroParisTech, Université Paris-Saclay, 78850, Thiverval-Grignon, France. [4]Metagenopolis, INRA, Université Paris-Saclay, 78350, Jouy-en-Josas, France.

[5]Maat-Pharma, Pharmaceutical Development, 69007, Lyon, France.

*catherine.juste@inra.fr

Supplementary Figures

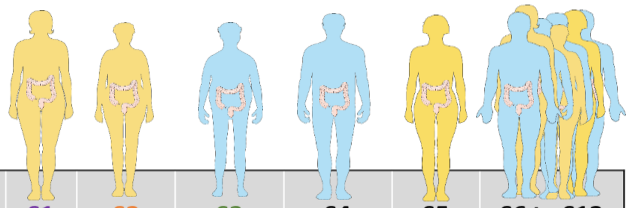

	Figures	Tables	Techniques	S1	S2	S3	S4	S5	S6 to S12
I. **Short-term storage - raw samples**	Fig. 1, 2	Table 1	16S rRNA + Metabolomic		X	X			
	Supp. Fig. S2		16S rRNA + Metabolomic		X	X			
II. **Short-term storage - cultures**	Fig. 3, 4	Table 2	16S rRNA + Metabolomic			X			
	Supp. Fig. S3		16S rRNA + Metabolomic			X			
III. **Diluents & Thawing**	Fig. 5	Table 3	16S rRNA	X	X	X			
	Fig. 6	Table 3	Metabolomic		X				
	Supp. Fig. S4		16S rRNA + Metabolomic	X	X	X			
IV. **Viability control of frozen transplants**	Fig. 7a		Flow cytometry		X	X	X	X	
	Fig. 7b		Flow cytometry		X		X		
V. **Viability of freeze-dried transplants**	Supp. Fig. S5		Flow cytometry	X	X	X			
VI. **Viability of residue-free microbiota**	Fig. 8		Flow cytometry		X	X	X	X	
	Supp. Fig. S6, S7	Supp. Table S1	Flow cytometry		X	X	X	X	
VII. **SOPs for viable transplants**	Supp. Fig. S8, S9		Flow cytometry						X

Supplementary Figure S1. Subjects who participated in the different steps of the study. Drawings at the top of the figure were obtained from the open access gallery Servier Medical Art (https://smart.servier.com), under the CC BY Unported 3.0 license (https://creativecommons.org/licenses/by/3.0)

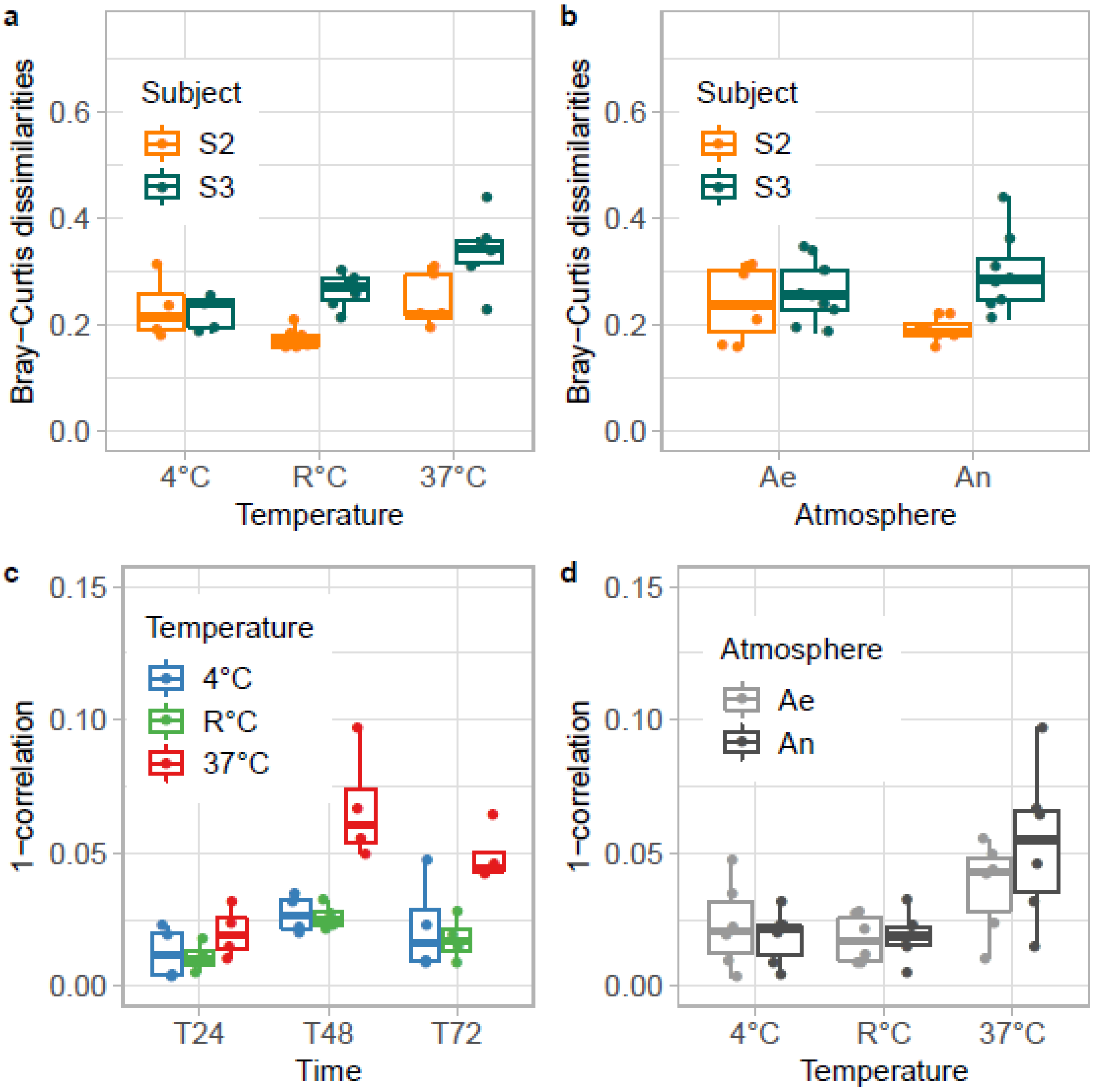

Supplementary Figure S2. Impact of short-term storage conditions on raw samples. Interaction temperature:donor (a) and atmosphere:donor (b) on microbial community shift from baseline, all other non-significant factors aggregated (see Table 1). Interaction time:temperature (c) and temperature:atmosphere (d) on metabolomics shift from baseline, all other non-significant factors aggregated (see Table 1). Baseline is the taxonomic or metabolomic profile in freshly voided feces.

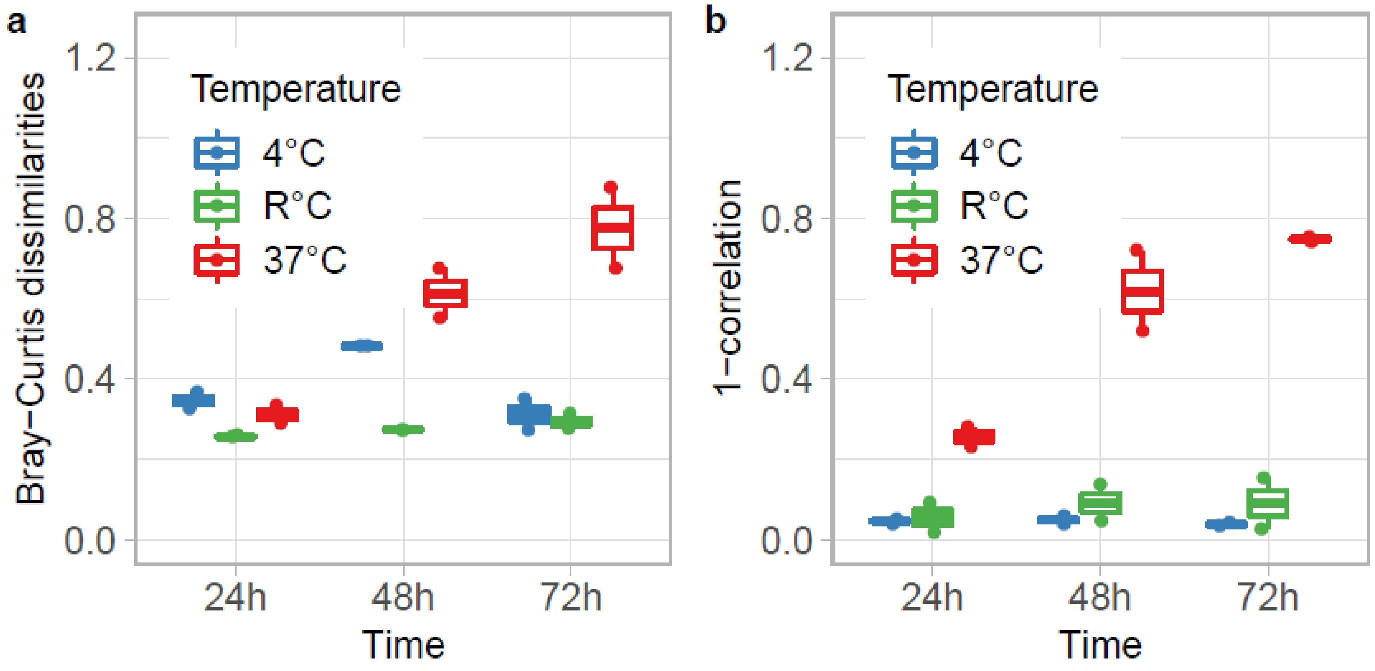

Supplementary Figure S3. Impact of short-term storage conditions on revivification potential of raw samples. Interaction time:temperature on taxonomic (a) and metabolomic (b) shift from baseline, all other non-significant factors aggregated (see Table 2). Baseline is the taxonomic or metabolomic profile in culture of freshly voided feces.

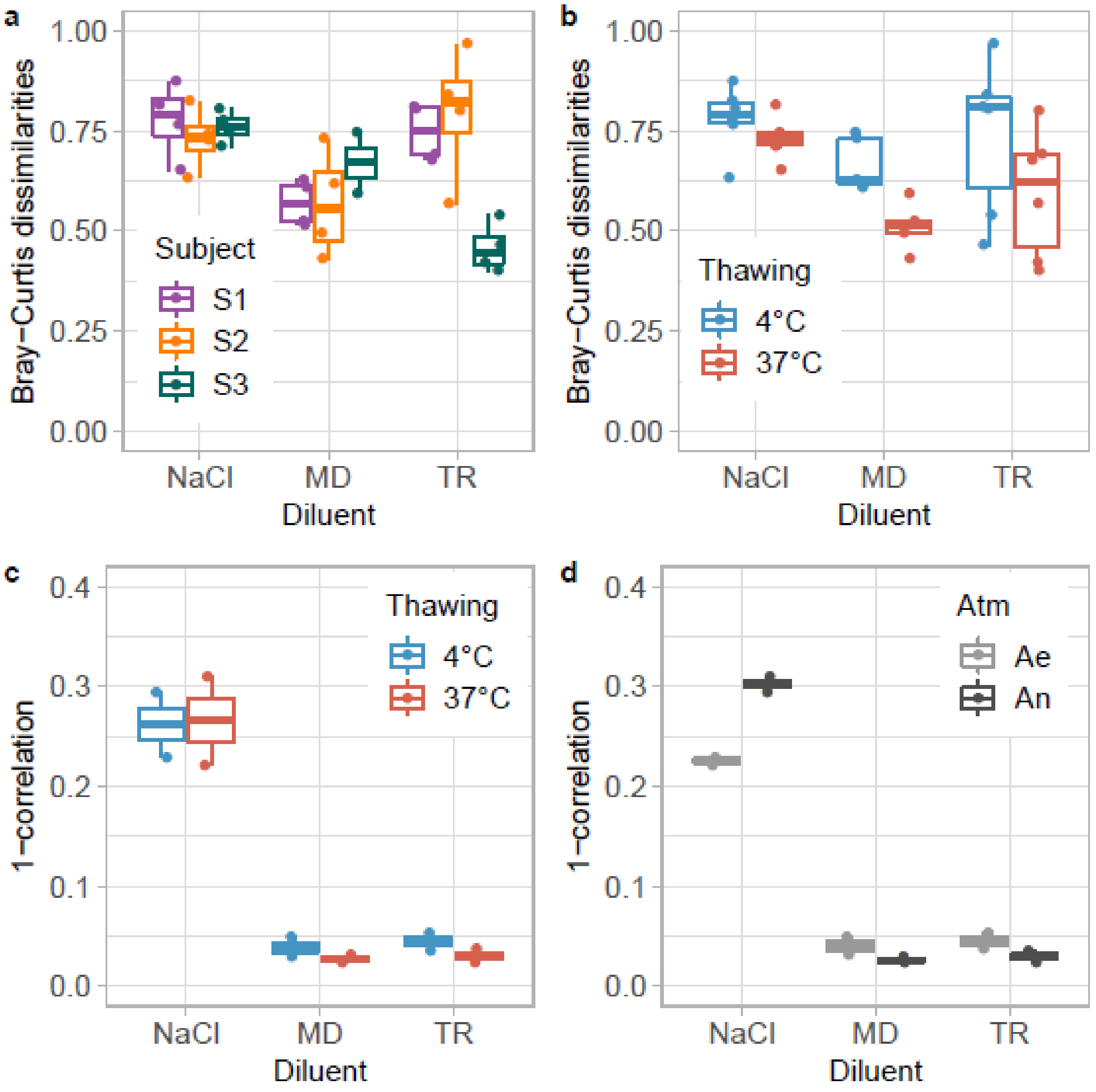

Supplementary Figure S4. Innovative conservative diluents and thawing conditions. Interaction diluent:donor (a) and diluent:thawing temperature (b) on microbial community shift from baseline in cultivated transplants, all other non-significant factors aggregated (see Table 4). Interaction diluent:thawing temperature (c) and diluent:atmosphere (d) on metabolomic shift from baseline in cultivated samples, all other non-significant factors aggregated (see Table 4). Baseline is the taxonomic or metabolomic profile in cultures of freshly voided feces.

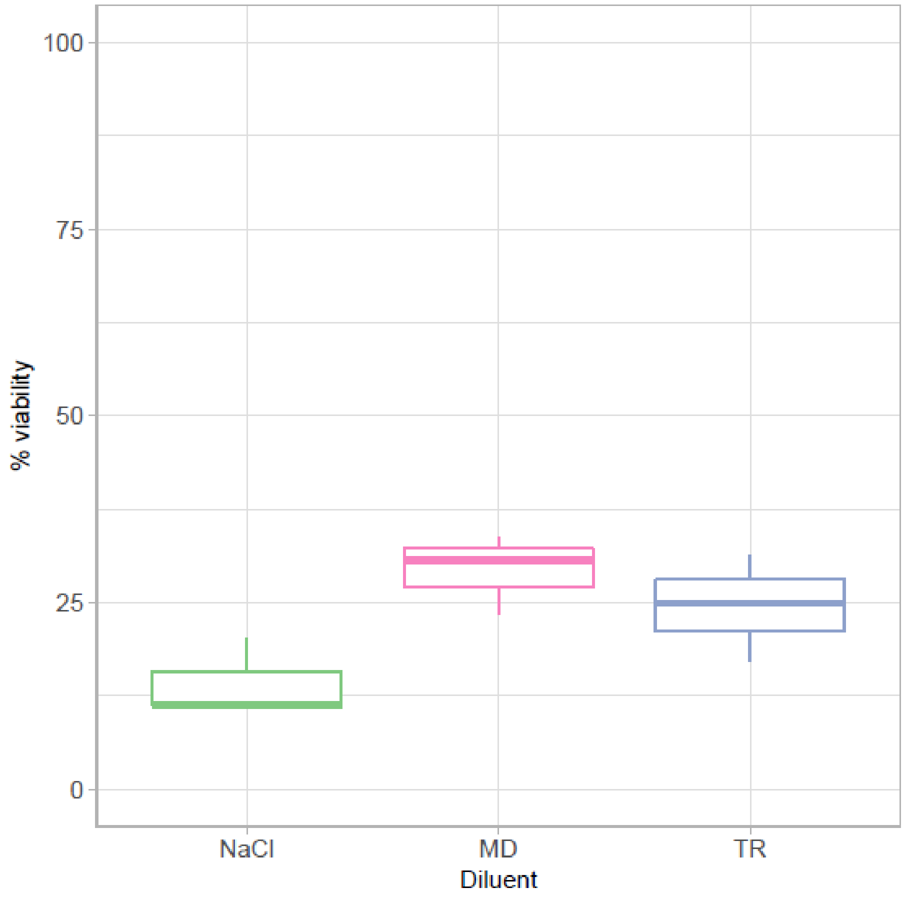

Supplementary Figure S5. Viability of freeze-dried transplants prepared in either NaCl or MD or TR.

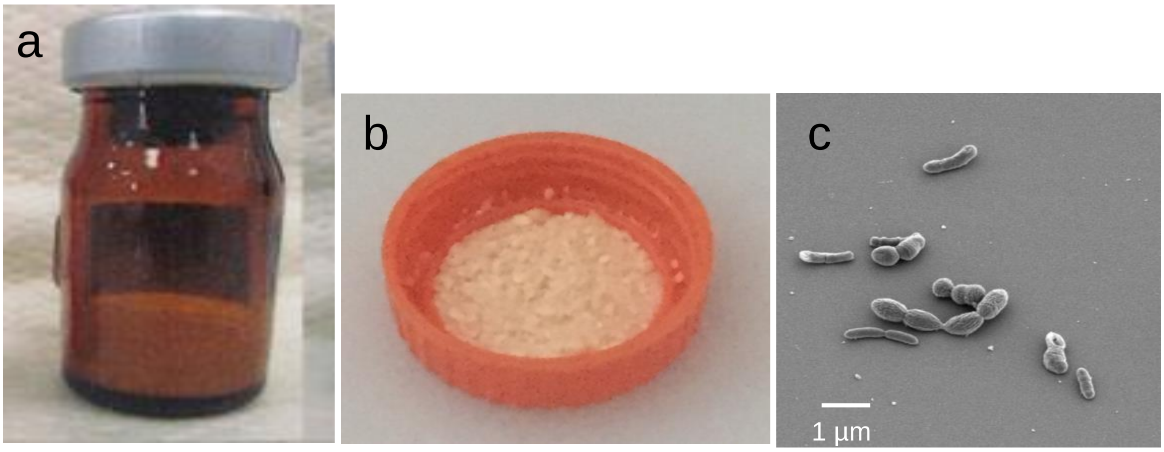

Supplementary Figure S6. Residue-free microbiota lyophilized in the newly developed diluent MD. (a) Stored at room temperature in sealed amber glass vials. (b) Macroscopic aspect of the vial content. (c) Microscopic aspect (scanning electron microscopy, MIMA2 platform, Inra-Jouy) of the vial content after rehydratation with reduced ultrapure water.

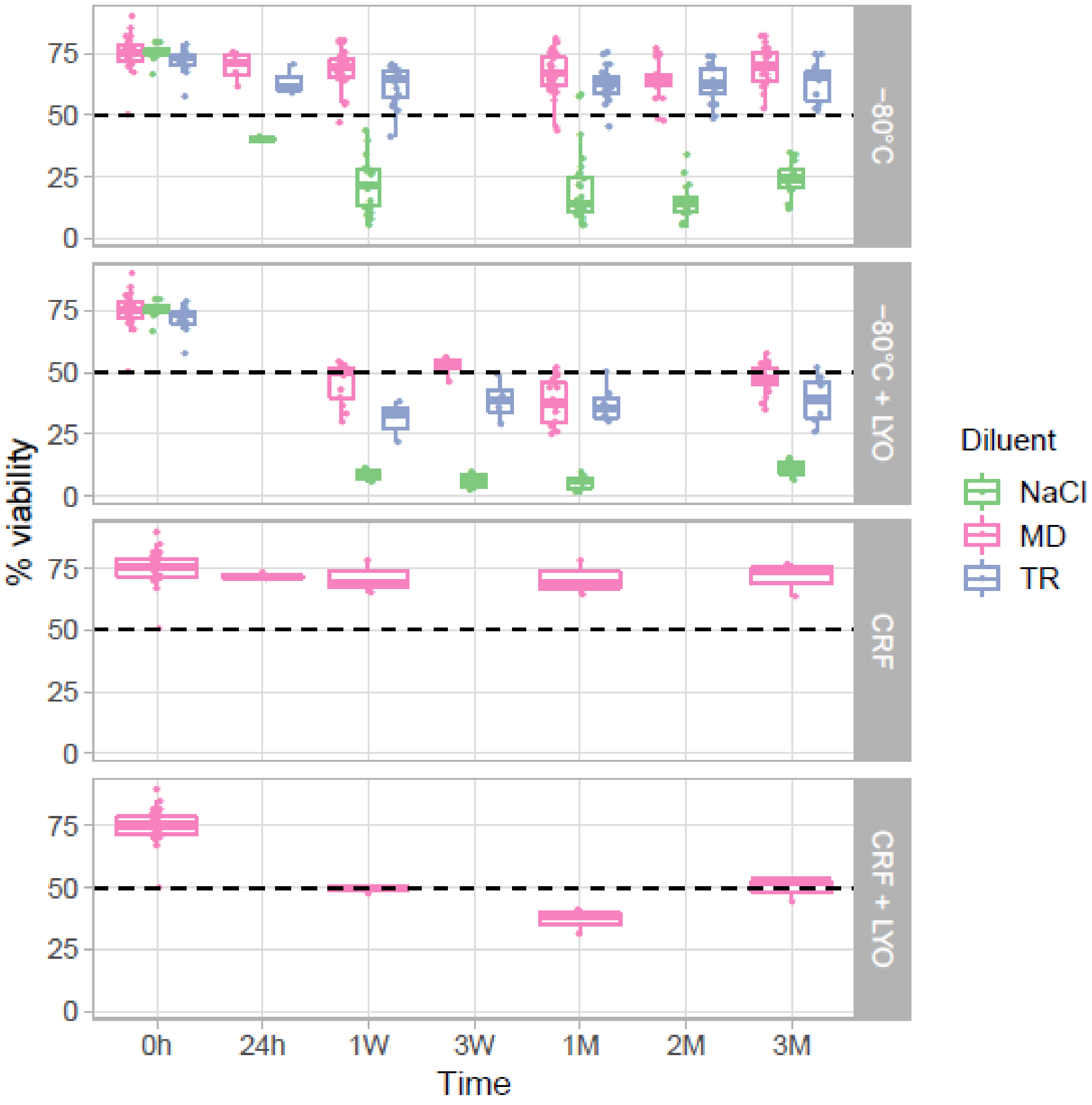

Supplementary Figure S7. Percent live bacteria in purified microbiota frozen in either at -80 °C standard freezer or at -100°C in a controlled-rate freezer (CRF), or further lyophilised (LYO) in either NaCl, or MD, or TR. At time intervals, aliquots of frozen samples were rapidly thawed and aliquots of freeze-dried samples reconstituted in a minimal volume of ultrapure water for live/dead tests by flow cytometry.

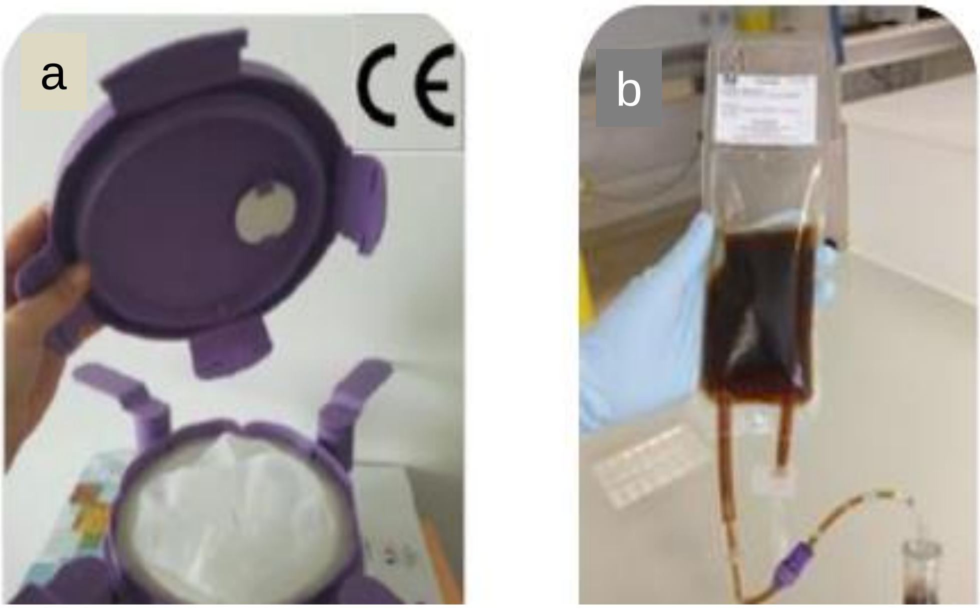

Supplementary Figure S8. Dilution, homogenization and filtration take place within the stool collection device itself (a). The obtained inoculum is directly transfered to 150-mL bags dedicated for FMT (b).

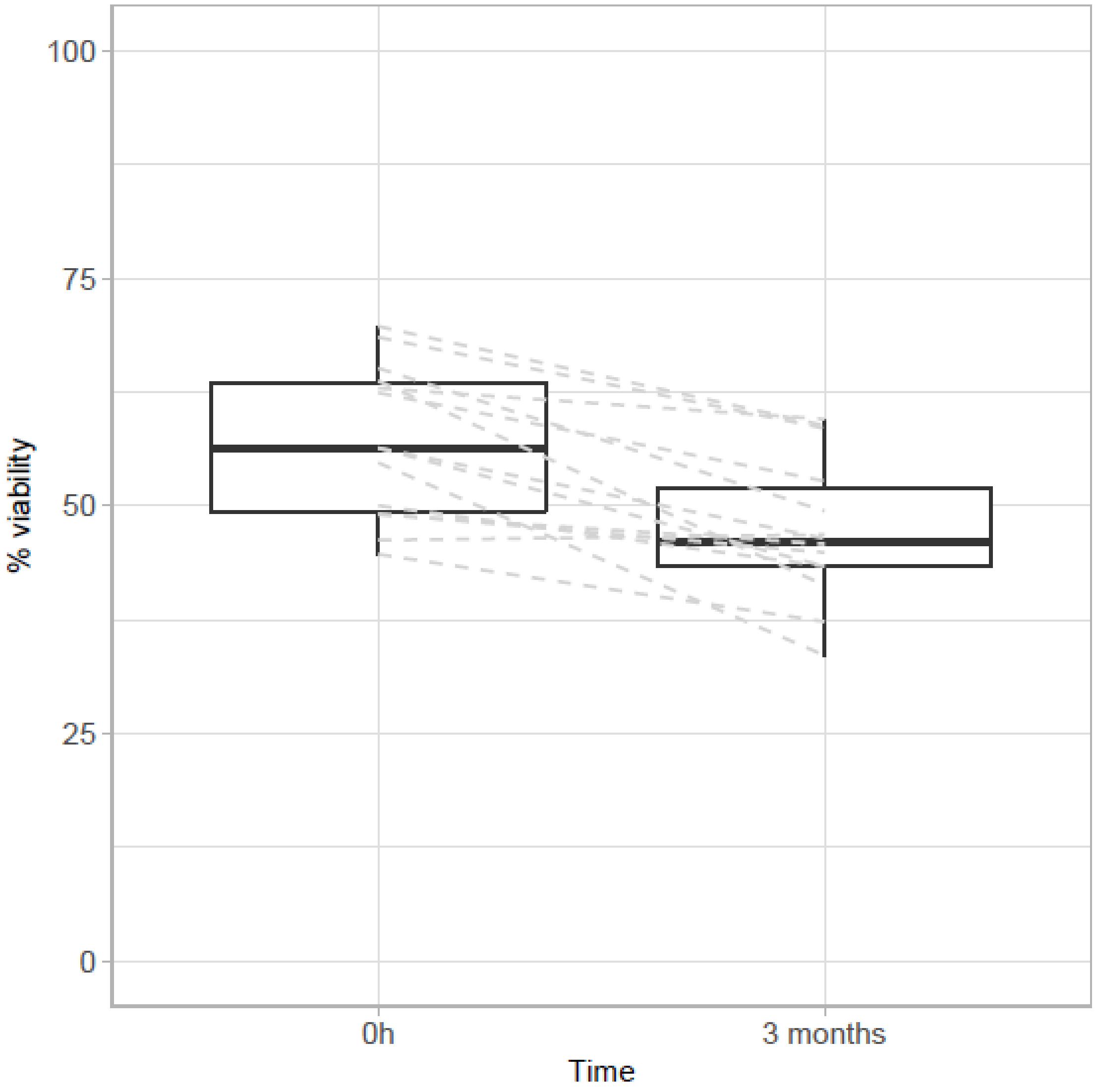

Supplementary Figure S9. Live/dead test by flow cytometry showing percent live bacteria in transplants prepared in the innovative diluent MD, frozen in a -80 °C standard freezer, and then rapidly thawed at 37°C after a three-month conservative period. Time 0 is before freezing.

Supplementary Table

Table S1. Results of the ANOVA. Viability of residue-free microbiota

	Estimate	Std. Error	t value	Pr(>|t|)
NaCl, freshly prepared	75.44131	2.16345	34.87081	2.07E-127
MD, freshly prepared	75.00554	1.58754	47.24641	2.46E-172
MD, CRF*	72.85584	2.48773	29.28605	1.82E-104
TR,freshly prepared	71.76808	2.16345	33.17296	1.31E-120
MD, -80°C	69.51807	1.13642	61.17276	2.68E-214
TR, -80°C	64.95831	1.33950	48.49444	1.95E-176
MD, CRF* + LYO**	46.22176	2.82879	16.33977	4.94E-47
MD, -80°C + LYO**	45.06342	1.31963	34.14858	1.55E-124
TR, -80°C + LYO**	38.09114	2.03477	18.72013	1.26E-57
NaCl, -80°C	23.18129	1.33523	17.36126	1.50E-51
NaCl:-80°C + LYO**	8.41309	2.11598	3.97597	8.22E-05
Subject S2	2.53676	2.54777	0.99568	3.20E-01
Subject S4	-4.00441	1.08065	-3.70554	2.38E-04
Subject S5	1.20575	1.55209	0.77685	4.38E-01
Subject S3	0.261909	1.09071	0.24013	8.10E-01
*-100 °C controlled-rate freezing (CRF)				
** freeze-dried				

Supplementary methods

Culture medium used for assessing the revivification potential. The Yeast Hemin Brain Heart Infusion (YHBHI) was prepared by dissolving 37 g Brain Heart Infusion, 5 g Yeast Extract, and 10 mL Hemin stock solution (50 mg Hemin dissolved in 1 mL 1 N NaOH, made up to 100 mL with distilled water, stored refrigerated) in reverse osmosis water to a final volume of 1 L, pH adjusted to 6.8 -7.2. The freshly prepared medium was dispensed into screw cap Kimble Kimax culture tubes (9.5 mL / tube). YHBHI tubes were immediately autoclaved at 121 °C for 15 min, and then entered the Freter chamber for equilibration at least 48 hours before use. Just before inoculation, each culture tube was enriched with 400 µL of a cocktail obtained by mixing equal volumes of stock solutions of 50% (w/v) Sodium L-ascorbate, 5% (w/v) L-Cysteine hydrochloride monohydrate, 10% (w/v) Cellobiose D-(+), and 10% (w/v) Maltose monohydrate, all solutions prepared in reduced water and filter-sterilized within the anaerobic chamber.

RNA extraction and microbial taxonomic (16S rRNA) profiling. DNA was digested using RNAse-free DNAse and DNA 10X reaction buffer supplied in the RQ1 RNAse free DNAse kit M6101 of Promega. The RNA extracts were suspended in 30 µL of Tris-HCl-EDTA buffer and stored at −80 °C. RNA integrity and quality was assessed by 2100 Bioanalyser instrument (Agilent Technologies) and Nanodrop instrument (Thermo Scientific). Then reverse transcription of the bacterial 16S rRNA into cDNA was performed with a high capacity cDNA RT-PCR (Applied Biosystems, V3fwd: 5’TACGGRAGGCAGCAG3’, V4rev: 5’GGACTACCAGGGTATCTAAT3’).

Microbiota composition was assessed by 454 pyrosequencing (innovative conservative diluent section) or Miseq sequencing (short term storage section) targeting the V3-V4 region of the bacterial 16S rRNA cDNA (V3fwd: 5'TACGGRAGGCAGCAG3', V4rev: 5'GGACTACCAGGGTATCTAAT3'). Sequencing was subcontracted to the company GenoScreen. Bioinformatic analyses were performed with FROGS pipeline. The same pipeline was applied for both sequencing technologies, excepted the preprocessing and the filters to remove rare OTUS (details below). Briefly, sequences were trimmed for barcodes, PCR primers, and less than 400 pb in length or N-containing sequences were removed. For 454 sequencing, we removed sequences with a quality below 50 on a sliding window or with a homopolymer over eight bases in length. Sequences were further clustered into OTUs (Operational Taxonomic Units or phylotypes) at 97% of identity using Swarm. Chimera were removed with Vsearch. We then applied filters to remove low abundance OTUs that are likely to be artefacts. Filters were adapted to sequencing depth: the threshold was 13 and 20 sequences/OTU for 454 and illumina sequencing, respectively. Resulting sequences were assigned to the different taxonomic levels, from phylum to genus using Blastall and the Silva database 123.

Preparation of Residue-free transplants. Extraction of microbial communities from the fecal matrix by flotation in a preformed continuous gradient was essentially as previously detailed[3] with some modifications. Briefly, we replaced Nycodenz® and Tris saline by the pharma grade products OptiPrep™ and Hepes, respectively, and we withdrew the detergent sodium deoxycholate from the stool diluent. In addition, faecal suspensions in OptiPrep were passed through a filter bag before being loaded under the preformed gradient. For purification of

microbiota by differential centrifugation, raw fecal material was diluted 50-fold in Hepes 10mM-NaCL 9g/L (50 mL / g feces) , and then passed through a filter bag before being centrifuged at low speed (300 × *g* for 5 min, 22 °C, in a swing-out rotor, Beckman Allegra X-30R centrifuge, rotor SX4400) for precipitation of coarse particles. Finally, the bacterial cells in the supernatant were spun down at 3,000 ´ *g* for 10 min at 22°C. All steps except the ultracentrifugation cycle of the gradients, were in the anaerobic chamber.

Gating live and dead bacterial populations. Gates of live and dead populations were drawn based on different samples that were divided into two fractions, one of which being untreated and the other treated with 70% isopropanol as a bactericide. Treated subsamples were prepared by the addition of 1.5mL of filter-sterilized 70% isopropanol on 50-100 µL of fecal suspension, followed by incubation for 1 h at room temperature, removal of the biocide by centrifugation (10 000 × *g* for 15 min), two washes of the pellet with 1.5mL normal saline and finally, decimal serial dilutions and staining as untreated subsamples. Interestingly, the same gates have proven to be valid for all samples, including control pure cultures, and were kept unchanged throughout the study. Results were expressed as percentages of live bacteria.

Supplementary references

1. Caporaso, J. G. *et al.* PyNAST: A flexible tool for aligning sequences to a template alignment. *Bioinformatics* **26,** 266–267 (2010).
2. Price, M. N., Dehal, P. S. & Arkin, A. P. FastTree 2 – Approximately Maximum-Likelihood Trees for Large Alignments. *PLoS One* **5,** (2010).
3. Juste, C. *et al.* Bacterial protein signals are associated with Crohn's disease. *Gut* **63,** 1566–1577 (2014).
